# Activation of *Xist* by an evolutionarily conserved function of KDM5C demethylase

**DOI:** 10.1038/s41467-022-30352-1

**Published:** 2022-05-11

**Authors:** Milan Kumar Samanta, Srimonta Gayen, Clair Harris, Emily Maclary, Yumie Murata-Nakamura, Rebecca M. Malcore, Robert S. Porter, Patricia M. Garay, Christina N. Vallianatos, Paul B. Samollow, Shigeki Iwase, Sundeep Kalantry

**Affiliations:** 1 Department of Human Genetics, University of Michigan Medical School, Ann Arbor, MI 48109-5618 USA; 2 Neuroscience Graduate Program, University of Michigan Medical School, Ann Arbor, MI 48109-5618 USA; 3 Department of Veterinary Integrative Biosciences, College of Veterinary Medicine and Biomedical Sciences, Texas A&M University, College Station, TX 77843-4458 USA; 4 Present Address: Department of Molecular Reproduction, Development and Genetics, Indian Institute of Science, Bangalore, Karnataka 560012 India; 5 Present Address: Department of Biology, University of Utah, Salt Lake City, UT 84112 USA

**Keywords:** Dosage compensation, Gene regulation

## Abstract

*XX* female and *XY* male therian mammals equalize X-linked gene expression through the mitotically-stable transcriptional inactivation of one of the two X chromosomes in female somatic cells. Here, we describe an essential function of the X-linked homolog of an ancestral X-Y gene pair, *Kdm5c*-*Kdm5d*, in the expression of Xist lncRNA, which is required for stable X-inactivation. Ablation of *Kdm5c* function in females results in a significant reduction in Xist RNA expression. *Kdm5c* encodes a demethylase that enhances *Xist* expression by converting histone H3K4me2/3 modifications into H3K4me1. Ectopic expression of mouse and human *KDM5C*, but not the Y-linked homolog *KDM5D*, induces *Xist* in male mouse embryonic stem cells (mESCs). Similarly, marsupial (opossum) *Kdm5c* but not *Kdm5d* also upregulates *Xist* in male mESCs, despite marsupials lacking *Xist*, suggesting that the KDM5C function that activates *Xist* in eutherians is strongly conserved and predates the divergence of eutherian and metatherian mammals. In support, prototherian (platypus) *Kdm5c* also induces *Xist* in male mESCs. Together, our data suggest that eutherian mammals co-opted the ancestral demethylase KDM5C during sex chromosome evolution to upregulate *Xist* for the female-specific induction of X-inactivation.

## Abstract (web summary)

Here the authors show eutherian mammals co-opted the histone demethylase KDM5C during sex-chromosome evolution to induce X-chromosome inactivation by upregulating Xist expression selectively in females.

## Introduction

X and Y chromosomes in therian mammals evolved from an ordinary pair of normally recombining autosomes^1–3^. As the Y chromosome formed, its ability to recombine with the X chromosome decreased incrementally and it lost much of its gene content^4^. Consequent to the loss of Y-linked genes in males, eutherian (placental) and metatherian (marsupial) females ultimately evolved X-chromosome inactivation, which silences genes along one of the two X chromosomes, thereby equalizing X-linked gene expression between the sexes^5^.

Although most X–Y homologs have been lost from the Y chromosome, some remain intact and functional. Moreover, the X-linked homologs of ancestral Y-linked genes that are broadly expressed almost invariably escape X-inactivation^6,7^. Escape from inactivation is thought to ensure similar combined expression of X–Y gene pairs between the sexes and is premised upon the homologs being functionally equivalent to one another^6,7^. Ancestral X–Y gene pairs, however, have been separated for ~100 million years and have accumulated amino acid substitutions due to lack of recombination^6,7^. Escape from X-inactivation of diverged X–Y gene pairs can lead to increased expression of the X-linked homolog in females vs. males and contribute to sexually dimorphic effects^8,9^, including potentially in X-inactivation in females^10,11^.

Stable X-inactivation in eutherians requires the Xist long noncoding RNA^12,13^, which is upregulated from and physically coats the future inactive X chromosome^14,15^. Xist RNA accumulation directly or indirectly recruits a suite of proteins that in turn stably silence X-linked genes^16–20^. How Xist RNA is preferentially upregulated in females, but not males, remains unclear^11^.

*Xist* and all of its transcriptional regulatory elements, including both *cis*-acting sequences and *trans*-acting factors, are believed to reside within the *X-inactivation center* (*Xic*) region of the X chromosome^21,22^. The *Xic* is thought to be both necessary and sufficient to induce X-inactivation^21,23,24^. One model of female-specific induction of X-inactivation relies on the sensing of the X chromosome complement via the physical pairing of the two Xs in *XX* cells through sequences within the *Xic*^25^. This coupling of the X chromosomes is proposed to result in a transvection-like mechanism that induces Xist RNA in *XX* females but not in *XY* males^26–28^. However, deletions of all candidate *Xic* elements involved in X chromosome pairing fail to prevent Xist upregulation from an X chromosome in differentiating female mouse embryonic stem cells (ESCs)^10,29^, which stochastically silence one of their two Xs during the process of random X-inactivation^30^. Moreover, physical separation of the two X chromosomes by tethering of one or both X chromosomes in female ESCs to the nuclear lamina nevertheless led to *Xist* induction upon differentiation of the ESCs^31^.

Another candidate mechanism underlying the female-specific induction of Xist RNA is the increased expression in *XX* females vs. *XY* males of *Xic*-encoded diffusible *Xist* activators that function in *trans*. The X-linked *Rnf12*/*Rlim* gene has been proposed to encode such a dose-dependent activator of *Xist*^32^. When ectopically expressed in *XY* male ESCs, *Rnf12*, which encodes an E3 ubiquitin ligase, can induce *Xist* in a subset of the cells^32^. *Rnf12* is also required to maintain Xist RNA expression during imprinted X-inactivation in mice^33^. Imprinted X-inactivation results in the silencing exclusively of genes on the paternally-inherited X chromosome in all cells of the preimplantation mouse embryo^34,35^. An *Rnf12* mutation on the maternally-derived X chromosome results in reduced Xist RNA expression from the paternal X chromosome in morula-stage female mouse embryos^33^. *Rnf12*, however, appears to be dispensable for Xist RNA induction during random X-inactivation that characterizes the epiblast lineage, which gives rise to all the somatic cell types and germ cells in the mouse embryo^34–37^. *Rnf12*^+/−^ and *Rnf12*^−/−^ female mouse embryonic epiblasts and differentiating female ESCs can upregulate Xist RNA expression, which suggests that alternate factors can induce Xist RNA in a dose-dependent manner during random X-inactivation^29,32,36,37^.

The *Xic*-linked *Ftx* and *Jpx*/*Enox* lncRNA-expressing loci have also been proposed to activate *Xist* expression in females^38–40^. However, deletions of *Ftx* and *Jpx*/*Enox* together with *Rnf12* on an X chromosome do not abrogate *Xist* induction in differentiating *XX* female ESCs but may lead to reduced *Xist* expression in *cis* from the mutant X chromosome^29^. These results suggest that *Ftx* and *Jpx*/*Enox* loci do not function as dose-dependent and diffusible *trans*-acting activators of *Xist* that induce X-inactivation selectively in females^41^. Other lncRNA-expressing loci in the *Xic* also appear to regulate *Xist* in *cis*, but not in *trans*^10,42^.

Our prior work suggested that *Xist* is preferentially induced in females at the onset of random X-inactivation by gene(s) that later escape X-inactivation^11^. We found that a disruption of the *Xist* antisense repressor *Tsix* ectopically induced *Xist* from the active *Tsix*-mutant X chromosome (*X*^Δ*Tsix*^) in differentiating pluripotent stem cells of both sexes in vivo and in vitro^10,11^. Interestingly, however, the ectopic expression of Xist RNA from the active *X*^Δ*Tsix*^ occurred in greater numbers of differentiating *X*^Δ*Tsix*^*X* female pluripotent embryonic epiblast cells and cultured epiblast stem cells (EpiSCs) compared to *X*^Δ*Tsix*^*Y* male cells^11^. EpiSCs are primed pluripotent stem cells^43,44^, which capture X-inactivation shortly after it has begun^10,11^. Since female EpiSCs harbor an inactivated X chromosome, we hypothesized that the increased dose in *X*^Δ*Tsix*^*X* females compared to *X*^Δ*Tsix*^*Y* males of one or more genes which escape X-inactivation underlie the more robust ectopic *Xist* induction in differentiating *X*^Δ*Tsix*^*X* female vs. *X*^Δ*Tsix*^*Y* male EpiSCs^11^. We further postulated that the higher expression of such genes in females vs. males was also responsible for Xist expression at the onset of X-inactivation in wild-type female epiblast cells^11^. The discovery and characterization of such factor(s) promised to explain the female-specific induction of random X-inactivation. Through in vivo and in vitro experiments, we reveal a role for the evolutionarily conserved X-inactivation escapee *Kdm5c*, which encodes a histone H3 di- and tri-methylated lysine 4 (H3K4me2 and H3K4me3) demethylase^45,46^, in the dose-dependent upregulation of *Xist* expression in eutherian mammals.

## Results

### *Kdm5c* is required for robust Xist RNA expression in the mouse epiblast lineage

We nominated the lysine demethylase KDM5C as a candidate activator of *Xist* for two primary reasons. First, *Kdm5c* is a part of an ancestral X–Y chromosomal gene pair that is conserved across therian mammals^6,7^. *Kdm5c* escapes X-inactivation in both mouse and human^47–50^ and exhibits an X-inactivation escapee chromatin profile in opossum^51^, a marsupial species, suggesting a dose-dependent and evolutionarily conserved function. Second, hypomethylation of H3K4 is associated with *Xist* induction^52,53^.

**Fig. 1:**
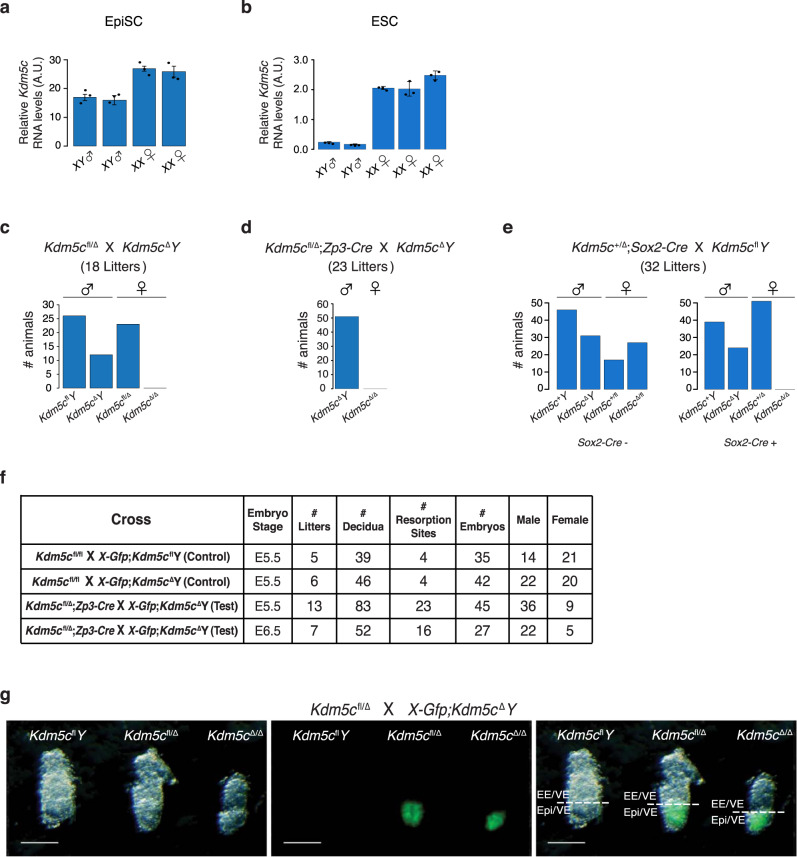
Lethality of *Kdm5c*^Δ/Δ^ female embryos. **a**, **b** Relative quantification of *Kdm5c* RNA levels in wild-type *XX* female and *XY* male EpiSCs (**a**) and ESCs (**b**) by RT-qPCR. Each bar in the graph represents an individual cell line analyzed by RT-qPCR in triplicate. Data are presented as mean $\pm$ standard deviation between technical replicates. **c**–**f** Quantification of liveborn mice (**c**–**e**) and recovery of embryonic day (**E**) 5.5 and 6.5 embryos (**f**). **g** Representative micrographs of E5.5 embryos. 13 litters were analyzed with similar results. Scale bars, 50 μm. Expression of a paternally-inherited *X-Gfp* transgene marks female embryonic epiblasts (Epi) but not extraembryonic ectoderm (EE) or visceral endoderm (VE) due to random X-inactivation in the epiblast lineage and imprinted X-inactivation of the paternal X-chromosome in extraembryonic lineages^60,61^. See also Supplementary Fig. 1a. Source data are provided as a Source Data file.

Because our prior work suggested that female EpiSCs express *Xist* activator(s) at a higher level than male EpiSCs^11^, we tested the relative levels of *Kdm5c* RNA in female vs. male EpiSCs by RT-qPCR. *Kdm5c* was consistently more highly expressed in female vs. male EpiSCs (Fig. 1a). We also quantified relative *Kdm5c* RNA levels in female vs. male ESCs. Whereas *XX* EpiSCs harbor an inactivated X chromosome, *XX* ESCs are in a pre-X inactivated state with two active X chromosomes and undergo X-inactivation only upon differentiation^30^. *Kdm5c* was also more highly expressed in female ESCs than in male ESCs (Fig. 1b). *Kdm5c* is therefore expressed more highly in female vs. male cells both after and prior to the initiation of X-inactivation, as we predict for an activator of *Xist*.

To test a female-specific role for *Kdm5c*, we took advantage of a *lox*p flanked (“floxed” or “fl”) conditionally mutant *Kdm5c* mouse strain^54^. Upon *Cre*-mediated recombination, the *Kdm5c*^fl^ allele generates a *Kdm5c*^Δ^ allele that lacks *Kdm5c* exons 11 and 12 (Supplementary Fig. 1a), which encode the enzymatic Jumonji-C (JmjC) demethylase domain and produces a barely detectable mutant protein^54,55^. A cross of *Kdm5c*^fl/Δ^ females with *Kdm5c*^Δ^*Y* males produced viable *Kdm5c*^Δ^*Y* males but not *Kdm5c*^Δ/Δ^ females (Fig. 1c). We next crossed *Kdm5c*^fl/Δ^ females harboring a *Zp3-Cre* transgene with *Kdm5c*^Δ^*Y* males. The expression of the *Zp3-Cre* transgene deletes floxed alleles in growing oocytes and thus ensures that the resulting embryos are depleted of any oocyte-derived KDM5C protein^56^, which may influence gene expression in the early embryo^57^. This cross also produced *Kdm5c*^Δ^*Y* males but no *Kdm5c*^Δ/Δ^ females (Fig. 1d).

Whereas embryonic epiblast cells of post-implantation female embryos undergo random X-inactivation, extraembryonic tissues maintain imprinted X-inactivation of the paternal X-chromosome that initiates in the preimplantation embryo^34,40,57,58^. We compared a requirement for *Kdm5c* in the epiblast vs. extraembryonic ectoderm by conditionally deleting *Kdm5c* using a *Sox2-Cre* transgene that efficiently excises floxed sequences in the nascent epiblast^59^. As with the crosses that generated constitutively mutant *Kdm5c*^Δ/Δ^ females (Fig. 1d), the epiblast-specific deletion of *Kdm5c* resulted in the absence of liveborn *Kdm5c*^Δ/Δ^ females (Fig. 1e).

To define when *Kdm5c*^Δ/Δ^ females were being lost, we isolated embryonic day (E) 5.5 and E6.5 post-implantation embryos from control and test crosses. In these crosses, the sires harbored a *Gfp* transgene on the X chromosome (*X-Gfp*), which is only transmitted to female progeny and thus permits visual sexing of the embryos^60,61^. Whereas the control crosses generated the expected distribution of female and male embryos, the test cross yielded fewer E5.5 and E6.5 *Kdm5c*^Δ/Δ^ female embryos compared to *Kdm5c*^Δ^*Y* male embryos (Fig. 1f). Moreover, the surviving *Kdm5c*^Δ/Δ^ E5.5 embryos were smaller relative to their heterozygous *Kdm5c*^fl/Δ^ female and *Kdm5c*^fl^*Y* male littermates (Fig. 1g).

**Fig. 2:**
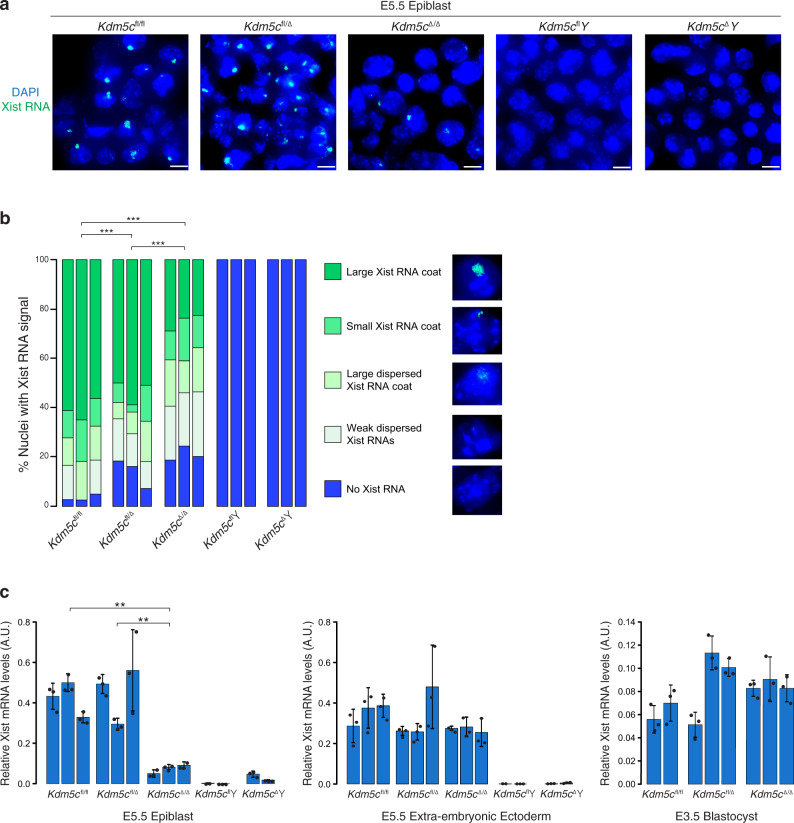
Reduced Xist RNA expression in *Kdm5c*^Δ/Δ^ female embryos. **a** Strand-specific RNA FISH detection of Xist RNA in representative E5.5 embryonic epiblast cells. Nuclei are stained blue with DAPI. Scale bars, 10μm. RNA FISH analysis was performed on 3 embryos/genotype with similar results. **b** Quantification of Xist RNA-coated nuclei in the E5.5 epiblast cells. Number of nuclei counted in each of the three embryos/genotype: *Kdm5c*^fl/fl^, *n* = 36, 77, and 80; *Kdm5c*^fl/Δ^, *n* = 76, 68, and 55; *Kdm5c*^Δ/Δ^, *n* = 128, 139, and 84; *Kdm5c*^fl^*Y*, *n* = 150, 200, and 100; *Kdm5c*^Δ^*Y*, *n* = 100, 93, and 85. *P* values, Chi-square test between female genotypes. *P ≤ 0.05; **P < 0.01; ***P < 0.001. See Supplementary Table 1 for Welch’s two-sided *t* test for all pairwise statistical comparisons. **c** Relative quantification of Xist RNA by RT-qPCR in E5.5 epiblasts, E5.5 extra-embryonic ectoderm, and E3.5 embryos. Each bar in the graph represents an individual E5.5 embryo fragment or E3.5 embryo analyzed by RT-qPCR in triplicate. Data are presented as mean $\pm$ standard deviation between technical replicates. *P* values, Welch’s two-sided *t* test on ΔCt values of biological replicates. *P ≤ 0.05; **P < 0.01; ***P < 0.001. Selected pairwise comparisons are shown. See Supplementary Table 2 for all pairwise statistical comparisons. See also Supplementary Fig. 1b. Source data are provided as a Source Data file.

From the results shown in Fig. 1, we surmised that the under-representation of E5.5 *Kdm5c*^Δ/Δ^ embryos may be due to defective random X-inactivation, which begins in the epiblast cells between E5.0-E5.25 stage of embryogenesis^10,62^. We therefore isolated E5.5 embryonic epiblasts and examined Xist expression by RNA fluorescence in situ hybridization (FISH) and RT-qPCR. Compared to *Kdm5c*^fl/fl^ and *Kdm5c*^fl/Δ^ cells, a significant number of E5.5 *Kdm5c*^Δ/Δ^ epiblast nuclei lacked large Xist RNA coats by RNA FISH (Fig. 2a). In addition, *Kdm5c*^Δ/Δ^ epiblasts showed a higher proportion of nuclei with dispersed or small Xist RNA signals (Fig. 2b and Supplementary Table 1). In accord with the RNA FISH data, RT-qPCR also revealed significantly reduced Xist RNA expression in E5.5 *Kdm5c*^Δ/Δ^ epiblasts compared to *Kdm5c*^fl/fl^ and *Kdm5c*^fl/Δ^ epiblasts (Fig. 2c and Supplementary Table 2). On average, Xist RNA expression in *Kdm5c*^Δ/Δ^ epiblasts was reduced by >80% compared to *Kdm5c*^fl/fl^ and *Kdm5c*^fl/Δ^ epiblasts.

To test if the deficiency in Xist RNA coating in *Kdm5c*^Δ/Δ^ epiblast was due to a failure of differentiation of the pluripotent epiblast progenitor cells, we concurrently assayed Xist RNA expression by FISH and the REX1 protein by immunofluorescence (IF) (Supplementary Fig. 1b). REX1 marks pluripotent epiblast progenitor cells and is rapidly downregulated when pluripotent cells differentiate^10^, concomitant with the onset of random X-inactivation^63^. As a control, we also conducted Xist RNA FISH and REX1 IF in E3.5 embryos, in which *Rex1* is expressed in the pluripotent epiblast progenitor cells of the inner cell mass^64^. Unlike E3.5 embryos, the E5.5 control and *Kdm5c*^Δ/Δ^ epiblast-enriched preparations were devoid of REX1 expression, indicating that they had undergone differentiation (Supplementary Fig. 1b).

We next tested Xist RNA expression in extraembryonic ectoderm cells of E5.5 embryos and in the preimplantation E3.5-stage embryos, both of which are subject to imprinted X-inactivation^34,40,57,58^. In contrast to the E5.5 epiblasts, Xist RNA levels in the three genotypes were comparable in female E5.5 extraembryonic ectoderm and E3.5 embryos (Fig. 2c and Supplementary Table 2). Xist RNA coating in *Kdm5c*^Δ/Δ^ and *Kdm5c*^fl/fl^ E3.5 embryos also appeared similar (Supplementary Fig. 1b). Together, these data demonstrate that *Kdm5c* is required for robust Xist RNA expression during random but not imprinted X-inactivation.

**Fig. 3:**
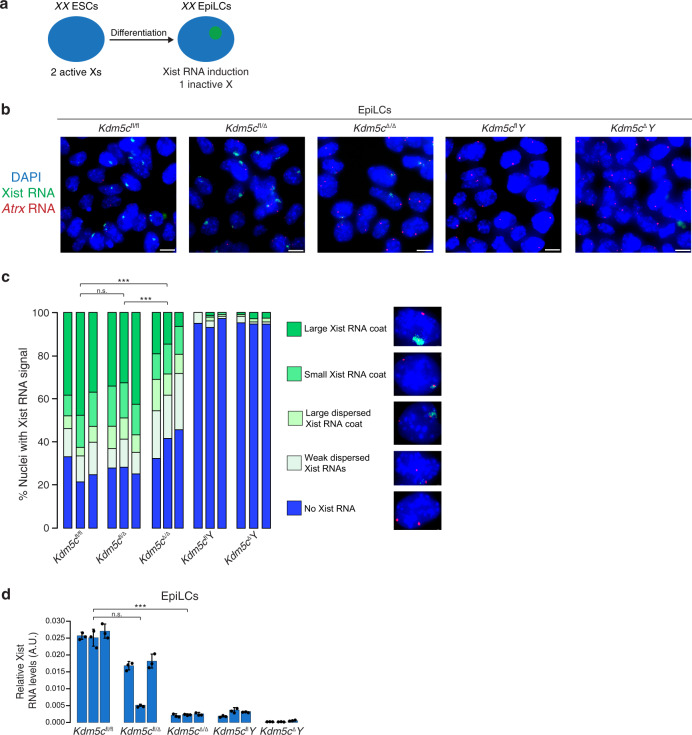
Reduced Xist RNA expression in *Kdm5c*^Δ/Δ^ female epiblast-like cells (EpiLCs). **a** Schematic of induction of X-inactivation upon differentiation of ESCs into EpiLCs. See also Supplementary Fig. 2a. **b** Detection of Xist RNA (green) by strand-specific FISH together with *Atrx* RNA (red) in representative EpiLCs. Scale bars, 10 μm. Three independent ESC lines were differentiated into EpiLCs/genotype with similar results. **c** Quantification of Xist RNA coating in EpiLCs. Xist RNA expression was quantified in five categories. Numbers of nuclei counted in EpiLCs differentiated from each of three independent ESC lines/genotype: *Kdm5c*^fl/fl^, *n* = 154, 122, and 113; *Kdm5c*^fl/Δ^, *n* = 107, 140, and 130; *Kdm5c*^Δ/Δ^, *n* = 145, 163, and 151; *Kdm5c*^fl^*Y*, *n* = 124, 230, and 111; *Kdm5c*^Δ^*Y*, *n* = 110, 138, and 114. *P* values, Chi-square analysis between female genotypes. n.s. not significant; *P ≤ 0.05; **P < 0.01; ***P < 0.001. See Supplementary Table 3 for Welch’s two-sided *t* test on all pairwise statistical comparisons. **d** Quantification of relative Xist RNA levels by RT-qPCR in the EpiLCs. Three independent ESC lines were differentiated into EpiLCs/genotype. Each bar in graph represents an individual ESC line differentiated into EpiLCs for each genotype. Data are presented as mean $\pm$ standard deviation between technical PCR replicates. *P* values, Welch’s two-sided *t* test on ΔCt values of biological replicates. n.s. not significant; *P ≤ 0.05; **P < 0.01; ***P < 0.001. See Supplementary Table 4 for all pairwise statistical comparisons. See also Supplementary Fig. 2b. Source data are provided as a Source Data file.

Epiblasts of surviving E5.5 *Kdm5c*^Δ/Δ^ embryos appeared smaller than that of *Kdm5c*^fl/Δ^ and *Kdm5c*^fl^*Y* littermates (Fig. 1g). To address the possibility that reduced Xist RNA expression in *Kdm5c*^Δ/Δ^ epiblast-enriched cells is a consequence of stunted development of the mutant embryos and to ensure quantitation of *Xist* induction in a pure population of cells of the epiblast lineage, we derived and analyzed *Kdm5c*^fl/fl^, *Kdm5c*^fl/Δ^, and *Kdm5c*^Δ/Δ^ female ESCs. Differentiation of *XX* ESCs into epiblast-like cells (EpiLCs) results in the stochastic inactivation of one X chromosome^10,30^ (Fig. 3a and Supplementary Fig. 2a). As in the E5.5 embryonic epiblast cells, *Kdm5c*^Δ/Δ^ EpiLCs showed fewer nuclei with large Xist RNA coats and a higher proportion of nuclei with dispersed or small Xist RNA signals compared to *Kdm5c*^fl/fl^ and *Kdm5c*^fl/Δ^ cells. (Fig. 3b, c and Supplementary Table 3). EpiLCs of all three genotypes showed downregulation of REX1, suggesting appropriate differentiation of the mutant ESCs (Supplementary Fig. 2b). The detection by FISH of biallelic expression of the X-linked gene *Atrx* demonstrates that the lack of Xist RNA coating in *Kdm5c*^Δ/Δ^ EpiLCs is not due to the loss of one of the two X chromosomes in the EpiLCs (Fig. 3b).

In agreement with the RNA FISH data, RT-qPCR also displayed significantly reduced Xist RNA levels in *Kdm5c*^Δ/Δ^ EpiLCs compared to *Kdm5c*^fl/fl^ EpiLCs and comparable to that found in *Kdm5c*^fl^*Y* and *Kdm5c*^Δ^*Y* male EpiLCs (Fig. 3d and Supplementary Table 4). On average, Xist RNA expression in *Kdm5c*^Δ/Δ^ EpiLCs was reduced by >90% compared to *Kdm5c*^fl/fl^ EpiLCs.

Moreover, the similar expression of Xist RNA in *Kdm5c*^Δ/Δ^ female and *Kdm5c*^fl^*Y* male EpiLCs suggests that Xist RNA induction in *Kdm5c*^Δ/Δ^ female cells reflects a basal level of Xist expression. Interestingly, *Kdm5c*^fl/Δ^ heterozygous EpiLCs expressed *Xist* at an intermediate level between *Kdm5c*^fl/fl^ and *Kdm5c*^Δ/Δ^ EpiLCs, suggesting a dose-dependent requirement for KDM5C in Xist RNA induction, as our hypothesis predicts.

### Ectopic expression of mouse and human KDM5C induces Xist RNA in male ESCs

**Fig. 4:**
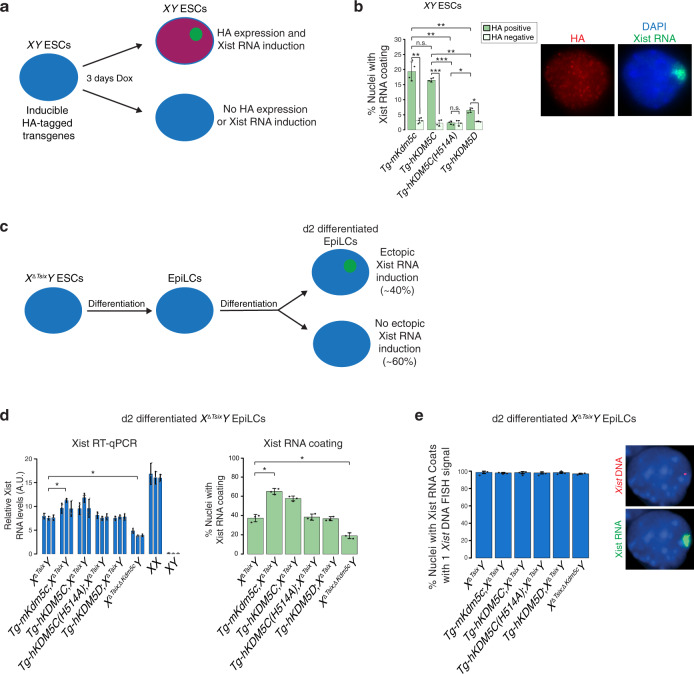
Ectopic KDM5C expression induces *Xist* in male ESCs. **a** Schematic of ectopic expression of HA-tagged transgenes in *XY* male ESCs. **b** Quantification of nuclei with Xist RNA coating in transgenic (*Tg*) *XY* male ESC lines (*n* = 3) expressing HA-tagged mouse KDM5C (*Tg-mKdm5c*; *n* = 292, 437, and 210); human KDM5C (*Tg-hKDM5C*; *n* = 154, 212, and 184); enzymatically inactive KDM5C (*Tg-hKDM5C(H514A)*;^45^ *n* = 292, 295, and 316); and, KDM5D (*Tg-hKDM5D*; *n* = 262, 222, and 232) proteins. Representative nuclei with IF detection of the HA epitope tag (red) coupled with FISH detection of Xist RNA (green) in the *XY* male ESCs. Data are presented as mean $\pm$ SEM. *P* values, Welch’s two-sided *t* test. n.s., not significant; *P ≤ 0.05; **P < 0.01; ***P < 0.001. See Supplementary Table 5 for all pairwise statistical comparisons. **c** Schematic of ectopic Xist RNA induction in day (d) 2 differentiated *X*^Δ*Tsix*^*Y* male ESCs. See also Supplementary Fig. 2c, d. **d** Quantification of ectopic Xist RNA expression by RT-qPCR (left) and Xist RNA-coated nuclei by RNA FISH (right) in d2 differentiated *X*^Δ*Tsix*^*Y* EpiLCs ectopically expressing mouse KDM5C (*Tg-mKdm5c*;*X*^Δ*Tsix*^*Y*); human KDM5C ^(^*Tg-hKDM5C*;*X*^Δ*Tsix*^*Y*); enzymatically inactive KDM5C (*Tg-hKDM5C(H514A)*;*X*^Δ*Tsix*^*Y*); KDM5D (*Tg-hKDM5D*;*X*^Δ*Tsix*^*Y*); and, *X*^Δ*Tsix*^*Y* EpiLCs lacking KDM5C (*X*^Δ*Tsix*;Δ*Kdm5c*^*Y*). For both the Xist RNA FISH and the Xist RT-qPCR data, three independent ESC lines/genotype were differentiated into d2 differentiated EpiLCs/genotype. For Xist RNA coating analysis, *n* = 100 nuclei per sample. Data are presented as mean $\pm$ SEM. For RT-qPCR, each sample was analyzed in triplicate. Data are presented as mean $\pm$ standard deviation between technical replicates. *P* values, Welch’s two-sided *t* test on ΔCt values of biological replicates. *P ≤ 0.05; **P < 0.01; ***P < 0.001. See Supplementary Table 6 for all pairwise statistical comparisons. **e** DNA FISH following RNA FISH demonstrates the presence of one X chromosome in nuclei with ectopic Xist RNA coating in the differentiated EpiLCs. *n* = 100 nuclei from three independent ESC lines differentiated into EpiLCs/genotype. Data are presented as mean $\pm$ SEM. See also Supplementary Fig. 3. Source data are provided as a Source Data file.

Having established that KDM5C is necessary for robust Xist RNA expression in *XX* female epiblast cells, we next tested the sufficiency of KDM5C in inducing Xist RNA by ectopically expressing KDM5C in *XY* male ESCs (Fig. 4a). Strikingly, mouse *Kdm5c* (*Tg*-*mKdm5c*) and human *KDM5C* (*Tg-hKDM5C*) transgene expression resulted in induction of Xist RNA in ~15-20% of *XY* male ESCs (Fig. 4b and Supplementary Table 5). A transgene harboring a point mutation that abolishes the demethylase function of KDM5C (*Tg-hKDM5C(H514A)*^45,55^) was not able to induce Xist RNA in the *XY* male ESCs (Fig. 4b and Supplementary Table 5). Ectopic expression of the closely related homolog, *KDM5D* (*Tg*-*hKDM5D)*, which maps to the Y chromosome and is itself capable of demethylating H3K4me2/3^45^, was also able to induce Xist RNA but at a level significantly lower than mouse or human KDM5C (Fig. 4b and Supplementary Table 5). Thus, KDM5C and KDM5D appear to be distinct in their quantitative capabilities to activate *Xist*.

### Dose-dependent enhancement of *Xist* by KDM5C

To test the dose-dependent activation of *Xist* by KDM5C, we next took advantage of the sensitized background of *X*^ΔTsix^*Y* ESCs. Some but not all differentiating *X*^ΔTsix^*Y* male EpiLCs ectopically express Xist RNA due to a truncation of the *Xist* antisense repressor RNA Tsix^11^ (Fig. 4c, Supplementary Fig. 2c, d, and Supplementary Fig. 3), which allowed us to assay changes in Xist RNA expression upon increased or decreased KDM5C expression. Compared to the parental *X*^ΔTsix^*Y* cells, differentiated *X*^ΔTsix^*Y* EpiLCs ectopically expressing mouse and human KDM5C displayed an increase in Xist RNA expression by RT-qPCR (*Tg-mKdm5c* *P* = 0.032; *Tg-hKDM5C P* = 0.054; Fig. 4d and Supplementary Table 6). Cells ectopically expressing the enzymatically inactive KDM5C or KDM5D proteins, on the other hand, exhibited Xist RNA expression levels similar to *X*^ΔTsix^*Y* cells (Fig. 4d). Reciprocally, the loss of KDM5C in *X*^Δ*Tsix*;Δ*Kdm5c*^*Y* cells significantly reduced ectopic Xist RNA expression relative to *X*^Δ*Tsix*^*Y* cells (*P* = 0.012; Fig. 4d and Supplementary Table 6). Xist RNA coating assayed by RNA FISH recapitulated the RT-qPCR data and DNA FISH confirmed the presence of a single X chromosome in these cells (Fig. 4d, e and Supplementary Table 6). These results are consistent with our hypothesis of a dose-dependent induction of *Xist* by KDM5C. Note that since *Tsix* is mutated in the *X*^Δ*Tsix*^*Y* EpiLCs^10,65,66^, these data rule out indirect induction of *Xist* by KDM5C-mediated suppression of *Tsix*.

### *Xist* enhancer activation by KDM5C

We next sought to test if KDM5C directly activates *Xist* expression. We, therefore, profiled the chromatin occupancies of KDM5C and H3K4me2 by chromatin immunoprecipitation followed by sequencing (ChIP-Seq) in the differentiating *X*^Δ*Tsix*^*Y* EpiLCs examined shown in Fig. 4. We used *X*^ΔTsix^*Y* male cells for ChIP analyses instead of *XX* female because in *XX* females, cellular mosaicism of X-linked gene expression due to random X-inactivation makes population measures such as ChIP-Seq and ChIP-qPCR incapable of distinguishing target molecule occupancy on the alleles of homologous X-linked genes on the inactive and active X chromosomes. In such a mosaic cell population, any incremental change in KDM5C and H3K4me2 at a given locus on the inactive X chromosome will be masked by the high levels of the mark at the homologous locus on the active X chromosome, which is enriched for the H3K4me2^67,68^.

**Fig. 5:**
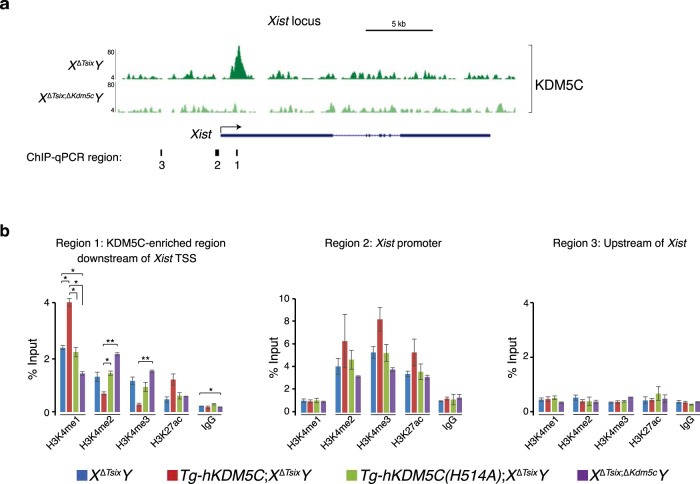
KDM5C upregulation of *Xist* expression via putative enhancer activation. **a** KDM5C enrichment at the *Xist* locus detected by ChIP-Seq. See Supplementary Table 7 for ChIP-seq read quantification. **b** ChIP-qPCR quantification of H3K4me1, H3K4me2, H3K4me3, and H3K27ac enrichment at the *Xist* locus. Locations of PCR amplicons are denoted beneath the browser views in (**a**). ChIPs were performed on day 2 differentiated EpiLCs generated from two independent ESC lines/genotype. qPCRs were performed in triplicate for each ChIP sample. Data are presented as mean $\pm$ SEM between ChIP replicates. Selected pairwise comparisons are shown. *P ≤ 0.05; **P < 0.01; ***P < 0.001, Welch’s two-sided *t* tests. See Supplementary Table 8 for all pairwise statistical comparisons. Source data are provided as a Source Data file.

We found that KDM5C is enriched ~1 kb downstream of the *Xist* transcriptional start site (TSS) (Fig. 5a and Supplementary Table 7). The KDM5C ChIP-Seq signal is absent in the *Kdm5c*-deficient *X*^Δ*Tsix*;Δ*Kdm5c*^*Y* cells, demonstrating its specificity. We next questioned how KDM5C-catalyzed demethylation of H3K4me2/3, which are enriched in transcriptionally active genes, could induce *Xist* expression. A potential answer lay in the KDM5C-catalyzed generation of H3K4me1 upon demethylation of H3K4me2/3^45^. H3K4me1 together with H3K27ac are enriched uniquely at enhancers^69,70^. We, therefore, tested H3K4me1/2/3 and H3K27ac occupancy at the KDM5C-enriched putative *Xist* enhancer element and two adjacent regions by ChIP-qPCR in the differentiating EpiLCs (Fig. 5b).

The KDM5C-enriched region downstream of the *Xist* TSS, Region 1, indeed displayed an enhancer-like chromatin signature of high H3K4me1 and lower H3K4me2/3 in differentiating *X*^ΔTsix^*Y* EpiLCs (Fig. 5b and Supplementary Table 8). Conversely, the *Xist* promoter region, Region 2, was decorated with higher H3K4me2/3 and lower H3K4me1. In Region 1, the putative *Xist* enhancer, ectopic expression of KDM5C but not the KDM5C H514A-mutant led to a further increase in H3K4me1 and H3K27ac along with a decrease in H3K4me2/3. In contrast, at the *Xist* promoter Region 2, which lacks KDM5C enrichment, we observed an increase in the active chromatin signatures H3K27ac but not H3K4me1 upon ectopic KDM5C expression (Fig. 5b and Supplementary Table 8). A region upstream of the *Xist* promoter, Region 3, did not show enrichment of the histone marks as observed in Regions 1 and 2. These data indicate that KDM5C directly promotes Xist RNA expression through the dose-dependent demethylation of H3K4me2/3 into H3K4me1 at an *Xist* enhancer element.

### An ancestral KDM5C function induces *Xist*

**Fig. 6:**
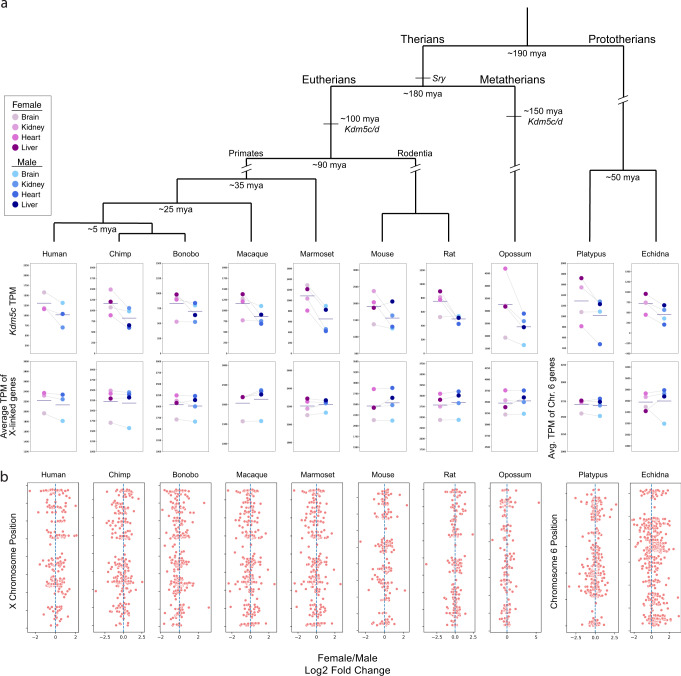
Phylogeny of *Kdm5c/d* and increased expression of *Kdm5c* in females vs. males in therian mammals. **a** Inferred phylogeny of *Kdm5c/d* during mammalian evolution^6,7,73^. Top row, *Kdm5c* transcript per million (TPM) levels in four tissues, as indicated, in females vs. males for seven eutherian species, one metatherian (marsupial) species (opossum), and two prototherian (monotreme) species (platypus and echidna). Bottom row, average TPM of all X-linked genes in females vs. males in the same tissues analyzed for *Kdm5c* expression. Platypus and echidna TPM averages are from Chromosome (Chr) 6, where KDM5C maps in those species^73^. The dark gray horizontal line in all plots represents the mean of all tissues. Light gray lines connect corresponding tissue types between males and females. **b** Distribution of female:male expression of genes across the therian X chromosome or monotreme Chr 6. Each dot is the log2 fold change of a gene’s expression in females vs males and was calculated from the average of that gene’s TPM in the brain, heart, kidney, and liver; human plot includes expression from the brain, heart, and kidney. See Supplementary Data 1–12 for sequencing data from each species.

The ability of human KDM5C to induce *Xist* in male mouse cells (Fig. 4) suggested that this property of KDM5C is evolutionarily conserved among eutherians. *Kdm5c* is an ancestral X chromosome gene that predates the eutherian and metatherian split and is intact in all therian species examined^6,7^. To gain insight into the extent to which the function of KDM5C is evolutionarily conserved, we first sought to test whether *Kdm5c* is expressed more highly in females vs. males across mammals. *Kdm5c* is predicted to be expressed more highly in therian females vs. males due to its likely escape from X-inactivation based on the presence of an active Y-linked *Kdm5d* homolog in almost all eutherians and metatherians examined^6,7^. To test this prediction, we measured the expression of *Kdm5c* in female and male tissues of seven eutherian species (human, chimp, bonobo, macaque, marmoset, rat, and mouse) and a metatherian (marsupial) species (opossum [*Monodelphis domestica*]), using prototherian (monotreme) species (platypus [*Ornithorhynchus anatinus*] and echidna [*Tachyglossus aculeatus*]) as outgroups^7,71^. Monotremes are the nearest extant relatives (sister taxon) of therian mammals but their X chromosomes do not undergo inactivation^6,7,72,73^. In the platypus and echidna, *Kdm5c* maps to Chromosome 6^73,74^, an autosome which is thought to represent the ancestral chromosome that gave rise to the therian sex chromosomes^73,74^, and *Kdm5d* is absent. In all eight therian species, the average *Kdm5c* expression across the tissues examined was higher in females vs. males (1.19–1.67-fold increase in females), compared to the average expression of X-linked genes generally (0.95–1.04-fold change in females vs. males) (Fig. 6a, b and Supplementary Data 1–12). Surprisingly, *Kdm5c* was also more highly expressed in females vs. males in both the platypus and the echidna (1.27- and 1.60-fold increase in females compared to the 1.01- and 0.99-fold female:male ratio of average Chr 6 gene expression for platypus and echidna, respectively), which suggests that the increased *Kdm5c* expression in therian females may have preceded the advent of therians.

**Fig. 7:**
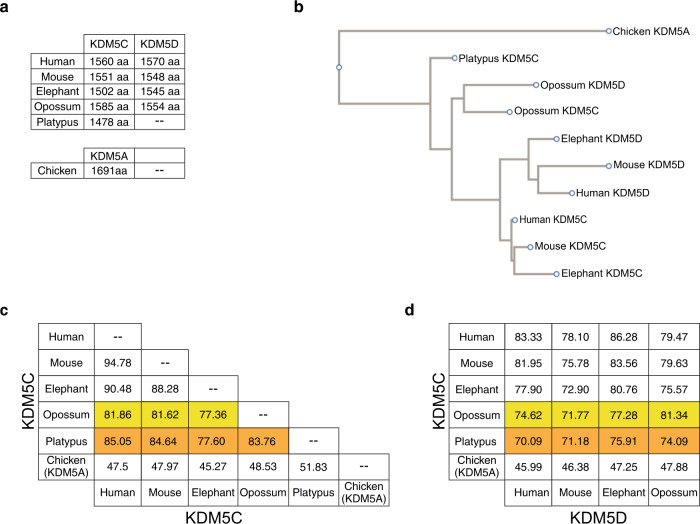
Divergence of KDM5C/D proteins during mammalian evolution. **a** Amino acid chain length of the KDM5C and KDM5D proteins. **b** Multiple sequence alignment phylogeny of KDM5C/KDM5D amino acid sequences using a neighbor-joining algorithm^101^. **c**, **d** Percent amino acid identity between therian, opossum, and platypus KDM5C proteins (**c**) and between KDM5C and KDM5D proteins (**d**).

We next examined the size and similarity of KDM5C and KDM5D proteins between three eutherian species (mouse, human, and elephant), opossum, and platypus (Fig. 7a, b). We found that opossum KDM5C displays a higher degree of amino acid identity to the three eutherian KDM5C proteins than to eutherian KDM5D (77.4–81.9% vs. 71.8–77.3%) (Fig. 7c, d). Platypus KDM5C similarly exhibits greater amino acid identity to eutherian KDM5C (77.6–85.0% identity) and opossum KDM5C (83.8% identity), and lower identity to eutherian and opossum KDM5D homologs (70.1–75.9% and 74.1% identity, respectively) (Fig. 7c, d).

**Fig. 8:**
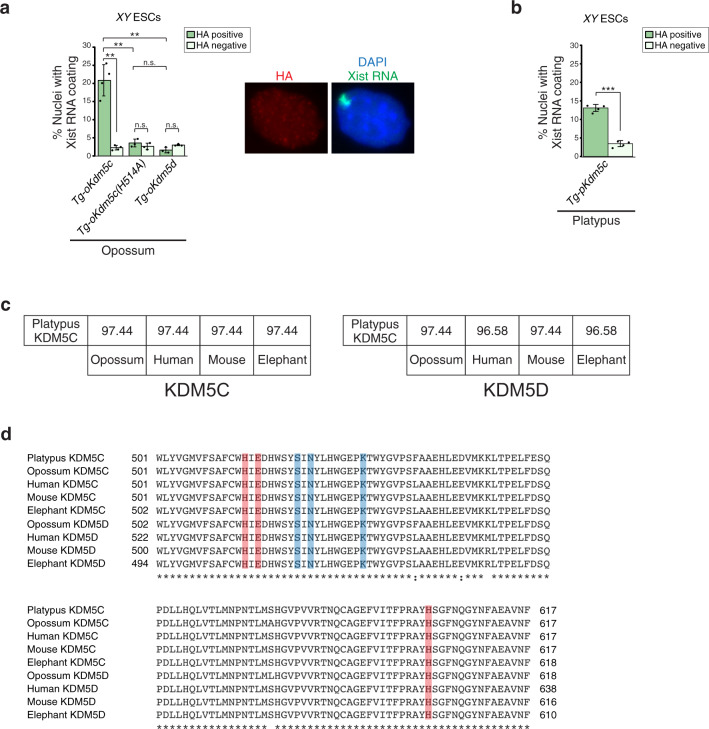
Opossum and platypus KDM5C activate *Xist* in male mouse ESCs. **a** Quantification of nuclei with Xist RNA coating in each of three independent transgenic (*Tg*) *XY* ESC lines expressing HA-tagged opossum KDM5C (*Tg-oKdm5c*; *n* = 209, 618, and 306); enzymatically inactive KDM5C protein (*Tg-oKdm5c(H514A)*; *n* = 196, 206, and 193); and, KDM5D (*Tg-oKdm5d*; *n* = 803, 562, and 636). Data are presented as mean $\pm$ SEM. *P* values, Welch’s two-sided *t* test. n.s., not significant; *P ≤ 0.05; **P < 0.01; ***P < 0.001. See Supplementary Table 9 for all pairwise statistical comparisons. **b** Quantification of nuclei with Xist RNA coating in each of three independent transgenic (*Tg*) *XY* ESC lines expressing HA-tagged platypus KDM5C (*Tg-pKdm5c*; *n* = 202, 178, and 525). Data are presented as mean $\pm$ SEM. *P* values, Welch’s two-sided *t* test. ***P < 0.001. See Supplementary Table 9 for pairwise statistical comparisons. **c** Percent amino acid identity between the catalytic JmjC domain of platypus KDM5C and therian KDM5C (left) or platypus KDM5C and therian KDM5D proteins (right). **d** Multiple sequence alignment of the demethylase JmjC domain of KDM5C and KDM5D proteins. The predicted iron-binding and the α-ketoglutarate-binding amino acids required for the catalytic activity of KDM5C/D are shown in orange and blue^55,76,102,103^, respectively. Source data are provided as a Source Data file.

The high level of identity among eutherian, metatherian, and prototherian KDM5C proteins prompted us to test whether *Kdm5c* from non-eutherian mammals could induce *Xist* in eutherians, e.g., mouse ESCs. Importantly, although X-inactivation occurs in all therian mammals, *Xist* has evolved only in eutherians and is absent in metatherians and prototherians^73,75^. We found that opossum KDM5C, but not KDM5D, can induce Xist RNA expression and coating in male mouse ESCs (Fig. 8a and Supplementary Table 9). Opossum *Kdm5c* containing the H514A point mutation, which is predicted to abolish the catalytic activity of KDM5C^45,55^, also resulted in failure to induce Xist RNA (Fig. 8a and Supplementary Table 9). *Xist* induction by both eutherian and metatherian KDM5C suggests that eutherian KDM5C has retained an ancestral therian capability which has been evolutionarily co-opted to induce *Xist* in eutherian mammals.

Platypus KDM5C, like opossum KDM5C, is more similar to therian KDM5C than to KDM5D, suggesting that platypus *Kdm5c* may also harbor the ancestral biochemical KDM5C function that underlies *Xist* induction in eutherians. We, therefore, tested the ability of platypus KDM5C to induce *Xist* by expressing platypus *Kdm5c* in male mouse ESCs. These transgene-expressing ESCs exhibited a significantly elevated percentage of nuclei with Xist RNA coating, indicating that the platypus *Kdm5c* transgene can indeed induce *Xist* in male mouse ESCs (Fig. 8b and Supplementary Table 9), albeit at a slightly lower level compared to mouse, human, and opossum *Kdm5c* transgenes (Figs. 4b and 8a). This implies that the properties of KDM5C that contribute to *Xist* induction in eutherian females date to at least the most recent common ancestor of all extant mammalian lineages.

In platypus KDM5C, the demethylating JmjC domain is 96–97% identical to the JmjC domain in therian KDM5C and KDM5D proteins, and the amino acid residues critical for co-factor binding for enzymatic activity are strictly conserved (Fig. 8c, d)^55,76^, suggesting that platypus KDM5C also functions as a H3K4me2/3 demethylase. To define the amino acids that differentiate KDM5C from KDM5D, we aligned mouse, human, elephant, opossum, and platypus KDM5C and KDM5D proteins^77^ (Supplementary Fig. 4a). Among eutherians, 45 amino acids are strictly conserved across the KDM5C proteins and have no overlap with those of KDM5D, across all species (Supplementary Fig. 4b). Among all therians (eutherians plus opossum), six amino acids are strictly conserved in KDM5C and have no overlap with KDM5D. Finally, considering all therians plus platypus, only four amino acids of KDM5C are fully conserved and fully distinct from those of therian KDM5D (prototherians lack *Kdm5d*).

## Discussion

In this study, we show that the histone H3K4me2/3 demethylase KDM5C is a primary driver of Xist RNA expression. KDM5C acts to enhance *Xist* RNA expression and does so in a dose- and the enzymatic-dependent manner in cells of the epiblast lineage that is subject to random X-inactivation. The biallelic and higher overall expression of *Kdm5c* in females vs. males links two primary steps that are thought to underlie the female-specific induction of random X-inactivation: sensing the X chromosome complement in the cell (“counting” step) and inducing expression of *Xist* (“initiation” step)^21,78^. All X chromosome sequence elements necessary and sufficient to induce X-inactivation are thought to reside within the *X-inactivation center* (*Xic*), which harbors the *Xist* locus^21,78^. *Kdm5c*, however, maps far outside of the *Xic*, near the telomeric end of the mouse X chromosome. Thus, the results herein suggest that elements outside of the *Xic* are required to induce X-inactivation through the regulation of *Xist*.

Upon the loss of KDM5C function, Xist RNA expression in female embryonic epiblasts and EpiLCs is significantly downregulated and approaches the levels found in male samples, suggesting that KDM5C is essential for the female-specific induction of *Xist*; however, residual Xist RNA expression in *Kdm5c*^Δ/Δ^ epiblasts and EpiLCs suggests that other *trans*-acting dose-dependent factors may also contribute to *Xist* induction in females. Based on the results of our previous study which postulated that genes that later escape X-inactivation induce *Xist* at the onset of the X-inactivation process^11^, we hypothesize that, in addition to KDM5C, other dose-dependent factors encoded by X-inactivation escapees may also contribute to *Xist* induction.

Recent work suggests that the autosomally encoded SPEN protein is required for Xist RNA induction^79^ (but, see also ref. ^80^). Since *Spen* is an autosomal gene, its expression levels are expected to be similar between females and males. SPEN induction of *Xist* may thus require the increased dose of an X chromosome-encoded factor in females vs. males that, for example, directly or indirectly recruits SPEN to activate *Xist* in females. In support of this notion, SPEN protein binds to and is enriched within the *Xist* locus^79,81^, including in the putative enhancer element at the 5’ end of *Xist* where KDM5C occupancy is also enriched. Similarly, KDM5C and other escapees may recruit or potentiate the functions of other *trans*-acting autosomally encoded factors proposed to activate *Xist*^82,83^.

X-inactivation is postulated to have evolved as a consequence of the deterioration of the Y chromosome^3^, which was once homologous to the X chromosome in the common ancestor of therian mammals^4^. By extension, the presence of active Y-linked genes is believed to have driven escape from inactivation of their X-linked homologs to adjust the dose of X–Y gene pairs between the sexes^84^. Our data instead support the notion that X–Y homologs can be functionally distinct, with the X-linked copy having evolved a female-specific function to initiate or contribute to X-inactivation. Escape from X-inactivation may therefore be evolutionarily driven in part by the requirement of the X-linked homologs in X chromosome dosage compensation.

As predicted from its escape from X-inactivation, in therians we found *Kdm5c* is more highly expressed in the tissues of females relative to males. Unexpectedly, though, *Kdm5c* expression was also higher in female than male prototherians, in which *Kdm5c* maps to an autosome and sex-specific inactivation phenomena are unknown. It thus appears that the basis for higher levels of KDM5C in females vs. males differs between therians and prototherians, with therian females achieving it through simultaneous expression of both *Kdm5c* alleles via escape from X-inactivation and prototherians likely through increased expression from each *Kdm5c* allele in females relative to that in males. These considerations suggest that higher expression of *Kdm5c* in females relative to males is a fundamental mammalian characteristic that could predate the therian/prototherian divergence and the advent of X-inactivation.

Of X–Y gene pairs, only *Sox3/Sry*, *Rbmx/Rbmy*, and *Kdm5c/Kdm5d* are conserved in all therian species examined^6,7^. Whereas Y-linked *Sry* triggers male sexual differentiation^85^ and *Rbmy* is inferred to regulate spermatogenesis^86^, X-linked *Kdm5c* is required for the female-specific upregulation of *Xist* expression in mice. *Kdm5c/d* is one of only two ancestral X–Y gene pairs, along with *Ube1x*/*y*, that have diverged independently, i.e., at different times during evolution, in eutherians and metatherians^6,7^. The independent divergence of *Kdm5d* from *Kdm5c* in eutherians and metatherians is unlikely to be due to chance and suggests selection for the survival of the X–Y gene pair in both therian lineages^6,7^. Together, the increased expression of *Kdm5c* in females vs. males across therians suggestive of X-inactivation escape, *Xist* induction in eutherian cells by eutherian, metatherian, and prototherian KDM5C, and the independent differentiation of *Kdm5d* from *Kdm5c* in eutherians and metatherians suggest that an evolutionarily conserved biochemical function of KDM5C has been recruited to help induce X-inactivation selectively in females in eutherians and potentially in metatherians as well. We cautiously speculate that KDM5C may contribute to X-inactivation induction in metatherian females by dose-dependent activation of the X-linked *Rsx* gene, which encodes an RNA with *Xist*-like expression and functional properties^87,88^.

## Methods

### Ethics statement

This study was performed in strict accordance with the recommendations in the Guide for the Care and Use of Laboratory Animals of the National Institutes of Health. All animals were handled according to the protocols approved by the University Committee on Use and Care of Animals (UCUCA) at the University of Michigan (protocol #PRO00004007 and PRO00006455).

### Mice

The generation of *Kdm5c*^fl^, *Tsix*^Δ^ (*Tsix*^AA2Δ1.7^), *X-Gfp*, *Zp3-Cre*, and JF1 strains have been described previously^54,56,60,66,89^. All mutant and transgenic strains were generated on the 129/Sv or background except for the *Kdm5c*^fl^ strain, which was generated on a mixed background. The expression of the *Zp3-Cre* transgene deletes floxed alleles in growing oocytes and thus ensures that the resulting embryos are depleted of any oocyte-derived maternal KDM5C protein^56^, which may influence X-inactivation patterns in the early embryo. We generated mutant embryos from a cross of *Kdm5c*^fl/Δ^;*Zp3-Cre* females with *Kdm5c*^Δ/Y^ males harboring the *X-Gfp* transgene (*X-Gfp*;*Kdm5c*^Δ/Y^). Mutant embryos were also generated from a cross of *Kdm5c*^fl/Δ^ females with *X-Gfp*;*Kdm5c*^Δ/Y^ males. *Kdm5c*^Δ/Δ^ embryos from both crosses showed similar phenotype (Fig. 1). The paternally transmitted *X-Gfp* transgene permits visual sexing of the embryos since it is only transmitted to female progeny^60,61^.

### Embryo dissections and processing

Pre-, peri-, and post-implantation stage embryos were isolated essentially as described previously^65^. Briefly, E3.5 embryos were flushed from the uterine limbs in 1X PBS (Invitrogen, #14200075) containing 6 mg/ml bovine serum albumin (BSA; Invitrogen, #15260037). The zona pellucida surrounding E3.5 embryos were removed through incubation in cold Acidic Tyrode’s Solution (Sigma, #T1788), followed by neutralization through several transfers of cold M2 medium (Sigma, #M7167). GFP fluorescence conferred by the paternal transmission of the *X-Gfp* transgene was used to distinguish female from male embryos, since only females inherit the paternal X chromosome. Isolated embryos were either lysed for RNA isolation in 100ul of Lysis/Binding buffer (Dynabeads mRNA Direct Purification Kit, ThermoFisher, #61012) or plated onto gelatin-coated glass coverslips in 0.25× PBS with 6 mg/ml BSA for immunofluorescence (IF)/RNA fluorescence in situ hybridization (FISH) staining. For plated embryos, excess solution was aspirated, and coverslips were air-dried for 15 min. After drying, embryos were permeabilized and fixed in 50 μl solution of 0.05% Tergitol (Sigma, #NP407) and 1% paraformaldehyde (Electron Microscopy Sciences, #15710) in 1× PBS for 10 min. Excess solution was tapped off onto paper towels, and coverslips were rinsed 3× with 70% ethanol and stored in 70% ethanol at −20 °C prior to RNA FISH. For isolation of post-implantation embryos (E5.5 and E6.5), individual implantation sites were cut from the uterine limbs and decidua were removed with forceps in 1× PBS/6 mg/ml BSA. Embryos were dissected from the decidua, and the Reichert’s membranes surrounding post-implantation embryos were removed using fine forceps. The visceral endoderm layer was removed by passing the embryo through a finely pulled glass Pasteur pipet. For separation of extraembryonic and epiblast portions of embryos, fine forceps were used to physically bisect the embryos at the junction of the epiblast and extraembryonic ectoderm. The epiblast was further distinguished by GFP fluorescence conferred by the paternally transmitted *X-Gfp* transgene; the transgene is mosaically expressed in the epiblast due to random X-inactivation but is silenced in the extraembryonic tissues because of imprinted X-inactivation of the paternal X^60,61,90,91^. Extraembryonic and embryonic epiblast cells were then separately either lysed for RNA isolation in 100 µl of Lysis/Binding buffer (Dynabeads mRNA Direct Purification Kit, ThermoFisher, #61012) or plated in 0.25× PBS with 6 mg/ml BSA onto gelatinized coverslips for IF/RNA FISH staining. Samples plated for IF/RNA FISH were fixed, permeabilized, and stored as described for E3.5 embryos.

### Derivation and culture of embryonic stem cell (ESC) lines

ESC lines were derived as previously described^30,92^. Briefly, individual E3.5 blastocyst are collected and plated on MEF cells in ESC derivation medium (Knockout DMEM (GIBCO, #10829-018), 20% knockout serum replacement (KSR; GIBCO, #A1099201), 2 mM l-glutamine (GIBCO, #25030), 1× nonessential amino acids (GIBCO, #11140-050), 1× penicillin–streptomycin (Gibco#15070-063), and 0.1 mM 2-mercaptoethanol (Sigma, #M7522) supplemented with 3 µM GSK3 inhibitor CHIR99021 (Stemgent#04-0004), 1 µM MEK inhibitor PD0325901 (Stemgent#04-0006), 1000 units/mL LIF (Millipore#ESG1106)) and cultured for 4–5 days. Then, individual blastocyst outgrowth is dissociated and cultured in 96 well containing MEF feeders. Mouse ESC colonies will be evident over the next 2–3 days. ESCs were cultured on mouse embryonic fibroblasts (MEFs) in knockout DMEM (GIBCO, #10829-018) with 15% knockout serum replacement (GIBCO, #A1099201), 5% ES cell qualified FBS (GIBCO, #10439024), 2 mM l-glutamine (GIBCO, #25030), 0.1 mM 2-mercaptoethanol (Sigma, #M7522), 1× nonessential amino acids (GIBCO, #11140-050), supplemented with 1000 units/ml LIF (Millipore #ESG1106).

For IF and/or RNA FISH, the cells were cultured on sterilized, gelatinized coverslips. The cells were permeabilized through sequential treatment with ice-cold cytoskeletal extraction buffer (CSK:100 mM NaCl, 300 mM sucrose, 3 mM MgCl_2_, and 10 mM PIPES buffer, pH 6. 8) for 30 s, ice-cold CSK buffer containing 0.4% Triton X-100 (Fisher Scientific, #EP151) for 30 s, followed with ice-cold CSK for 30 s. After permeabilization, cells were fixed by incubation in 4% paraformaldehyde for 10 min. Cells were then rinsed 3× in 70% ethanol and stored in 70% ethanol at −20 °C prior to IF and/or RNA FISH.

See Supplementary Data 13 for descriptions of all cell lines used in this study.

### Differentiation of ESCs into epiblast-like cells (EpiLCs)

ESCs were differentiated into EpiLCs as described^30^. To convert ESC to EpiLC, ESC lines were grown in 2i culture conditions (N2B27 medium [50% DMEM/F12 (GIBCO, #11330-032), 50% neurobasal media (GIBCO, #21103-049), 2 mM l-glutamine (GIBCO, #25030), 0.1 mM 2-mercaptoethanol (Sigma, #M7522), N2 supplement (Invitrogen #17502048), B27 supplement (Invitrogen #17504-044)], supplemented with 3 μM GSK3 inhibitor CHIR99021 (Stemgent #04-0004), 1 μM MEK inhibitor PD0325901 (Stemgent #04-0006), and 1000 units/ml LIF (Millipore #ESG1106)) in gelatin-coated tissue culture dishes for four passages^93,94^. Following the four passages, cells were cultured in N2B27 medium supplemented with 10 ng/ml FGF2 (R&D Systems, #233-FB-025) and 20 ng/ml Activin A (R&D Systems, #338-AC-005) in fibronectin (15 μg/ml) (Sigma #F1141)-coated tissue culture dishes for 48 hrs. For *X*^ΔTsix^*Y* cells, after EpiLC conversion the cells were further differentiated by continuing culture in N2B27 medium without FGF2 and Activin A for an additional 48 h. For IF and/or RNA FISH, cells were permeabilized, fixed, and CSK processed as described above for ESCs.

See Supplementary Data 13 for descriptions of all cell lines used in this study.

### Derivation and culture of epiblast stem cell (EpiSC) lines

EpiSCs were derived as described^30^ from E3.5 or E5.5 embryos. For the derivation of the EpiSC lines, whole E3.5 embryos or E5.5 epiblast layers were plated on MEF cells in EpiSC medium (Knockout DMEM (GIBCO, #10829-018) supplemented with 20% knockout serum replacement (KSR; GIBCO, #A1099201), 2 mM l-glutamine (GIBCO, #25030), 1× nonessential amino acids (GIBCO, #11140-050), and 0.1 mM 2-mercaptoethanol (Sigma, #M7522), 10 ng/ml FGF2 (R&D Systems, #233-FB-025)) and cultured for 3–4 days to form a large EpiSC colony. EpiSC colonies were then manually dissociated into small clusters using a glass needle and plated into 1.9-cm^2^ wells containing MEF feeders in EpiSC medium. EpiSCs were passaged every third day using 1.5 mg/ml collagenase type IV (GIBCO, #17104-019) with pipetting into small clumps.

See Supplementary Data 13 for descriptions of all cell lines used in this study.

### RNA fluorescence in situ hybridization (FISH)

RNA FISH with double-stranded (ds) and strand-specific (ss) probes was performed as previously described^90,95^. The dsRNA FISH probes were made by randomly priming DNA templates using BioPrime DNA Labeling System (Invitrogen, #18094011). The ssRNA FISH probes were made using the Invitrogen MAXIscript Kit (Invitrogen, #AM1326). ss*Xist* probes were generated from templates as previously described^65,96^. Probes were labeled with Fluorescein-12-dUTP or -UTP (Invitrogen) or Cy3-dCTP or –CTP (GE Healthcare). Labeled probes from multiple templates were precipitated in a 0.3-M sodium acetate solution (Teknova, #S0298) (for dsRNA FISH probe) and 0.5-M ammonium acetate solution (for strand-specific RNA FISH probe) along with 300 μg of yeast tRNA (Invitrogen, #15401-029) and 150 μg of sheared, boiled salmon sperm DNA (Invitrogen, #15632-011). The solution was then spun at 21,130 rcf for 20 min at 4 °C. The pellet was washed consecutively with 70% ethanol and 100% ethanol. The pellet was dried and re-suspended in deionized formamide (ISC Bioexpress, #0606). The probe was denatured by incubating at 90 °C for 10 min followed by an immediate 5 min incubation on ice. A 2× hybridization solution consisting of 4× SSC, 20% Dextran sulfate (Millipore, #S4030), and 2.5 mg/ml purified BSA (New England Biolabs, #B9001S) was added to the denatured solution. All probes were stored at −20 °C until use.

For analysis of Xist RNA coating in *XY* male cells that ectopically express various *Kdm5c/d* transgenes (Figs. 4a and  8a, b), Xist RNA FISH was performed with a dsRNA FISH probe as well as ssRNA FISH probe. Results with both probes were concordant and were combined for the quantification.

Cells or embryo fragments mounted on coverslips were dehydrated through 2 min incubations in 70%, 85%, 95%, and 100% ethanol solutions and subsequently air-dried. The coverslips were then hybridized to the probe overnight in a humid chamber at 37 °C. The samples were then washed 3× for 7 min each while shaking at 39 °C with 2XSSC/50% formamide, 3× with 2× SSC, and 2x with 1× SSC. A 1:250,000 dilution of DAPI (Invitrogen, #D21490) was added to the third 2× SSC wash. Coverslips were then mounted on slides in Vectashield (Vector Labs, #H-1000).

### DNA FISH

DNA FISH probes were prepared as described for double-stranded RNA FISH probes^11,95^. The BAC template used for *Xist* DNA FISH is RP24-287F13 (Children’s Hospital of Oakland Research Institute). After RNA FISH, cells were washed with 1× PBS three times and then incubated in PBS for 5 min at room temperature. The cells were then re-fixed with 1% (wt/vol) PFA containing 0.5% (vol/vol) Tergitol and 0.5% (vol/vol) Triton X-100 for 10 min at room temperature. The cells were next dehydrated through an ethanol series (70%, 85%, and 100% ethanol, 2 min each) and air-dried for 15 min. The cells were then treated with RNase A (1.25 μg/μl) at 37 °C for 30 min. The cells were again dehydrated through the ethanol series as described above. The samples were then denatured in a prewarmed solution of 70% formamide in 2× SSC on a glass slide stationed on top of a heat block set at 95 °C for 11 min followed immediately by dehydration through a −20 °C-chilled ethanol series (70%, 85%, 95%, and 100% ethanol, 2 min each). The cells were then air-dried for 15 min followed by probe hybridization overnight at 37 °C. The following day, the samples were washed twice with prewarmed 50% formamide/2× SSC solution at 39 °C and 2× with 2× SSC, 7 min each.

### Immunofluorescence (IF)

Cells mounted on glass coverslips were washed 3X in PBS for 3 min each while shaking. Coverslips were then incubated in blocking buffer consisting of 0.5 mg/ml BSA (New England Biolabs, #B9001S), 50 μg/ml yeast tRNA (Invitrogen, #15401-029), 80 units/ml RNAseOUT (Invitrogen, #10777-019), and 0.2% Tween 20 (Fisher, #BP337-100) in 1× PBS in a humid chamber for 30 min at 37 °C. The samples were next incubated with primary antibody diluted in blocking buffer for 1–3 h in the humid chamber at 37 °C. Anti-REX1 antibody (Thermo Scientific, #PA5-27567) was used at 1:150 dilution and anti-HA antibody (Cell Signaling, #C29F4) was used at 1:800 dilution. The samples were then washed 3× in PBS/0.2% Tween 20 for 3 min each while shaking. After a 5 min incubation in blocking buffer at 37 °C in the humid chamber, the samples were incubated in blocking buffer containing a 1:300 dilution of fluorescently conjugated secondary antibody (Invitrogen, Alexa Fluor 555, #A31572 for HA antibody; Alexa Fluor 647, #A31573 for REX1 antibody) for 30 min in the humid chamber at 37 °C, followed by three washes in PBS/0.2% Tween 20 while shaking for 3 min each. The samples were then processed for RNA FISH.

### RT-qPCR

Total RNA from cells was isolated from TRIzol (Life Technologies, #15596-018) according to the manufacturer’s instructions. mRNA from embryos was purified using Dynabeads mRNA direct Purification Kit (ThermoFisher, #61012) according to the manufacturer’s instructions. RT-qPCR was performed using SYBR Green-based relative quantification method on an Eppendorf Realplex Mastercycler (software version 2.2.0.84). The housekeeping gene *Gapdh* was used as an internal control for data normalization. Control reactions lacking reverse transcriptase for each sample were also performed to rule out genomic DNA contamination. RT-qPCR was carried out using the SYBR Green PCR Mastermix (Kapa Sybr Fasr One-Step Universal kit #KK4651 and/or Luna Universal One-Step RT-qPCR Kit #E3005L). The following primers were used for RT-qPCR: *Kdm5c*: forward TTTGGCAGCGGTTTCCCTGTCAGT, reverse AAGACCATTCCCACATACAGCC; *Rex1*: forward CCAGGTTCTGGAAGCGAGTT, reverse GCTTGAGGACACTCCAGCAT; RT-qPCR of *Xist* on *XY* male EpiSCs, *XX* female EpiSCs, and *Tsix*-mutant *XY* male EpiLCs: forward CAAGAAGAAGGATTGCCTGGATTT, reverse GCGAGGACTTGAAGAGAAGTTCTG. RT-qPCR of *Xist* on all other samples: forward CTCCCAGCATTACTGAGAAATG, reverse CCAGGCAATCCTTCTTCTTGAG. *Gapdh:* forward TGTGTCCGTCGTGGATCTGA, reverse CCTGCTTCACCACCTTCTTGA. *P* values for all RT-qPCR results were calculated using Welch’s two-sample *t* tests. RT-qPCR data is presented for each biological replicate. Error bars represent the standard deviation between technical replicates of multiple PCR reactions.

### Analysis of RNA-Seq data

Three independent ESC lines were differentiated into EpiLCs for each genotype (male *Kdm5c*^fl^*Y*, *Kdm5c*^Δ^*Y*; female *Kdm5c*^fl/fl^, *Kdm5c*^Δ/Δ^). The EpiLCs were then homogenized in TRI Reagent BD (Sigma) and subjected to total RNA isolation using the RNEasy Mini Kit (Qiagen). Ribosomal RNA was depleted from total RNA using the RiboMinus Eukaryote Kit v2 for RNA-Seq (Life Technologies). Libraries were prepared using Direct Ligation of Adapters to First-strand cDNA as described previously^85^. Multiplexed libraries were pooled in approximately equimolar ratios and purified from a 1.8% TBE-agarose gel. Libraries were sequenced on the Illumina HiSeq2500 platform, with single-end 50 base pair reads, according to standard procedures. Detailed information on reverse transcription, adapter design, ligation, and subsequent steps has been described previously^85^. Raw reads were demultiplexed and filtered according to the standard Illumina analysis pipeline. Reads from sequencing libraries were mapped to the mm9 build of the mouse genome using Tophat 2^86,87^ (version 2.1.0), allowing up to one mismatch, and only uniquely mapped reads were analyzed further. Bigwig files normalized to the read depth were used to confirm the lack of reads from deleted KDM5C exons.

### Expression plasmids and lentiviral transduction in ESCs

*mKdm5c*, *hKDM5C*, *hKDM5D*, *oKdm5c*, and *oKdm5d* were cloned from cDNAs into the Gateway Entry vector (pDONR221, Invitrogen). The *pKdm5c* cDNA sequence (Accession: XP_028923236) with the AttL1 and AttL2 recombination sequence at the 5´ and 3´ end, respectively was custom synthesized (Gene Universal Inc.). The *hKDM5C(H514A)* and *oKdm5c(H514A)* mutant cDNAs were generated by PCR-based mutagenesis. The sequence of all cDNAs was verified via Sanger sequencing. To generate the plasmids for stable overexpression in *X*^∆Tsix^*Y* male ESCs, the cDNAs were cloned into an in-house generated *PGK* promoter-driven Strep-tag II lentiviral vector by Gateway LR recombination. To generate doxycycline-inducible constructs used for overexpression in *XY* male ESCs, the cDNAs were cloned into a *PGK* promoter-driven HA-tag lentiviral vector pLIX-402 (Addgene plasmid # 41394) by Gateway LR recombination. For the production of lentivirus, 50–70% confluent HEK293T cells in a 10-cm tissue culture plates were co-transfected with 3.33 μg lentiviral construct, 2.5 μg psPAX2 packaging plasmid, and 1 μg pMD2.G envelope plasmid using TransIT-293 (Mirus). Lentiviral particles were collected 48 h post transfection. The lentivral particles were then concentrated using LentiX (Clontech #631231). Concentrated lentivirus was transduced to ESCs with 10 μg/ml Polybrene (Millipore, #TR-1003-G). After 72 h, the transduced cells were selected with 3 μg/ml Puromycin (Sigma, #P8833-25MG) and subcloned to generate clonal cell lines. For inducible overexpression, cells were treated with 2 µg/ml doxycycline (Sigma) for 3 days. Media were changed every 24 h with fresh doxycycline.

### ChIP

ChIP was performed as previously described^54^, with a minor modification, where we sonicated the chromatin using truChIP sonicator (Covaris). Two independent *X*^∆Tsix^*Y*, *X*^∆Tsix;∆Kdm5c^*Y*, or *Tg-mKdm5c*;*X*^∆Tsix^*Y* ESC lines were differentiated for 2 days (2d) beyond the epiblast-like cell (EpiLC) stage for ChIP with the exception that KDM5C ChIP was performed on one *X*^∆Tsix;∆Kdm5c^*Y* 2d differentiated EpiLC sample. To achieve optimal inter-sample normalization of ChIP efficiency, *Drosophila* chromatin (Abcam #58083, 100 ng), which contains fly-specific histone variant H2Av, was spiked in and subsequently immunoprecipitated with 0.4 μg anti-H2Av antibody (Active Motif #39715) according to the manufacturer’s instructions. Two to five million EpiLCs were lysed and incubated with 3 μg of one of the following antibodies: H3K4me1 antibody (Abcam #8895); H3K4me2 antibody (Abcam #7766); H3K4me3 antibody (Abcam #8580); H3K27ac antibody (Active motif #39135); rabbit IgG antibody (Jackson Immuno Research #011-000-003); or 10 μg of an in-house anti-KDM5C antibody^54^. DNA was purified by phenol/chloroform extraction and eluted using QIAquick purification kit (QIAGEN#28104).

### ChIP-Seq

ChIP-Seq libraries were sequenced on the Illumina HiSeq2500 platform to generate 50 bp single-end reads. Raw reads were demultiplexed and filtered according to the standard Illumina analysis pipeline. Reads from sequencing libraries were then mapped to the mouse (mm9) and fruit fly (dm6) genome assemblies using Bowtie^97^ (version 1.2.2) allowing up to two mismatches. Only uniquely mapped reads were used for analysis.

For KDM5C ChIP-Seq analysis, peaks were called using MACS2 software (version 2.1.0.20140616)^98^ using input BAM files for normalization, with filters for a *q*-value <0.1 and a fold enrichment greater than 1. This analysis yielded 98 peaks on the X chromosome for d2 differentiated *X*^∆Tsix^*Y* EpiLC samples; 1 peak for the *X*^∆Tsix;∆Kdm5c^*Y* d2 EpiLC sample. For visualization in the Integrated Genome Viewer, reads were extended by 180 bp, and bigwig files were generated with coverage normalized using the number of mapped reads to the *Drosophila* genome (reads mapped per reference genome per million reads)^99,100^.

### ChIP-qPCR

ChIP was performed as above. All qPCR reactions were performed using SYBR Green-based relative quantification method on an Eppendorf Realplex Mastercycler (software version 2.2.0.84). The following primers were used: *Xist* TSS downstream region (Region 1): forward CACAACCCCGCAAATGCTAC, reverse CGGCTATTCTCGAGCCAGTT; *Xist* promoter region (Region 2): forward ACTATGAGCGTAAGCCCACC, reverse CATGTATCGCTCCGCGTCTT; *Xist* upstream region (Region 3): forward GATCCATTCCATTAATACGAGCACT, reverse AGTTCACACTCCTCTGAGACCC.

### Fluorescent western blotting

Protein lysates were made in RIPA buffer (50 mM Tris-HCl, 1% NP40, 0.25% Na-deoxycholate, 150 mM NaCl) with PMSF (Sigma, #P7626) and protease inhibitor cocktail (Roche, #11873580001). Lysates were mixed with Laemmli sample buffer and boiled for 10 min prior to loading on polyacrylamide gels. Resolved proteins were transferred onto PVDF membranes (iBlot 2 PVDF, Invitrogen #IB24002) following the manual instructions for 7 min (iBlot 2 Dry Blotting System, Invitrogen #IB21001). Membranes were then blocked by Intercept Blocking Buffer (LI-COR, #927-70001) for 1 h at room temperature and incubated in the anti-Strep-Tag II (GenScript, #A01732-100; 1:1000 dilution) and anti-KDM5C (Abcam, #194288; 1:1000 dilution) primary antibodies together overnight at 4 °C. Appropriate Li-COR IRDye series (680LT, #926-68020/800CW, #926-32211) secondary antibodies were used (1:20,000 dilution). Blots were scanned using the Odyssey CLx Imager (Li-COR Biosciences) following the manufacturer’s instructions. Results were analyzed using Image Studio (Version 5.2). Transgenic proteins levels were normalized to KDM5C.

### Microscopy

Images of all stained samples were captured using a Nikon Eclipse TiE inverted microscope with a Photometrics CCD camera. The images were analyzed after deconvolution using NIS-Elements software (version 5.20.02, Build 1453). All images were processed uniformly.

### KDM5C/KDM5D sequence analysis

Amino acid sequences were obtained from the Ensembl database and from Cortez et al., 2014^7^. Human KDM5C accession NP_004178 human KDM5D accession NP_001140177 mouse KDM5C accession XP_006528832 mouse KDM5D accession NP_035549 elephant KDM5C ensemble transcript ID ENSLAFT00000027692.1 (https://useast.ensembl.org/Loxodonta_africana/Transcript/Summary?db=core;g=ENSLAFG00000000303;r=scaffold_85:1408730-1440791;t=ENSLAFT00000027692), elephant KDM5D accession JAC06693 opossum KDM5C accession GAQH01000002 opossum KDM5D accession JAC06620 platypus KDM5C accession XP_028923236 and chicken KDM5A accession XP_416379 Multiple sequence alignment was performed using ClustalW^77^ (version 2.1). MSAViewer^101^ (version 1.12.0) tree tool was used to create a rooted phylogenetic tree with branch lengths according to the Neighbor-Joining algorithm. Amino acid positions for the JmjC domains were acquired from the NCBI Conserved Domain Database^102^. Iron and α-ketoglutarate-binding sites were acquired from UniProt for human KDM5C and the human amino acid positions were compared to the other species^55,76,103^.

### Analysis of *Kdm5c* expression in therian and platypus females vs. males

Raw RNA-Seq fastq files were obtained from the NCBI Sequence Read Archive. RNA-seq data for human, chimp, bonobo, macaque, mouse, opossum, and platypus are available under GEO accession number GSE30352^71^. RNA-seq data for rat and marmoset are available under GEO accession number GSE50747^7,104^. Echidna RNA-seq data is available under project accession number PRJNA591380^73^. The brain and cerebellum data were combined into one “brain” sample. One male and one female sample of each brain, heart, kidney, and liver were processed in each species. The human dataset did not include a female liver sample, and so the liver was left out of the human analysis. Reads were mapped to the latest assembly reference genome for a given species using STAR (v2.5.2a), and reads were counted using HTseq (Python v2.7). Genes in a sample were only included in the analysis if they contained at least 10x read coverage. A gene was included if at least one tissue type met these criteria in male and female samples. For *Kdm5c* expression plots, *Kdm5c* TPM (transcripts per kilobase per million RNA-Seq reads) was calculated in each tissue (brain, heart, kidney, and liver), and TPM values for each tissue were plotted. For average X chromosome plots (or Chromosome 6 for platypus and echidna), TPM values of all genes were averaged in each tissue. Average TPM values for each of the four tissues were plotted. For dot plots, the TPM values from each tissue were averaged for each gene. Tissue averages for each gene were used to calculate Log2 fold change between females and males and plotted against the gene’s chromosome position. Gene length, for TPM calculations, was acquired from the RefSeq annotation for each species. Statistical analysis could not be performed due to a lack of biological replicates. See Supplemental Tables 6–17.

### Statistical analysis

Welch’s two-sided *t* test was performed using Microsoft Excel. Chi-square analysis of RNA FISH was performed using R (version 4.1.2). All exact *P* values are provided in Supplementary Tables 1–9.

### Reporting summary

Further information on research design is available in the Nature Research Reporting Summary linked to this article.

## Supplementary information

Supplementary Information

Description of additional Supplementary File

Supplementary Data 1-12

Supplementary Data 13

Reporting Summary

## Data Availability

The data that support this study are available from the corresponding author upon reasonable request. Sequencing data generated for this study have been submitted to the NCBI Gene Expression Omnibus (GEO; http://www.ncbi.nlm.nih.gov/geo/) under accession number GSE96797. Data in Fig. 6 were analyzed from published data available under accession numbers GSE30352 and GSE50747 and project accession number PRJNA591380. All samples used are listed in Supplementary Data 1 and raw data from these analyses are available in Supplementary Data 2–12. Source data are provided with this paper.

## Source data

Source Data
