# Supplementary material for: Activation of Xist by an evolutionarily conserved function of KDM5C demethylase
Source: Nat Commun. 2022 May 11;13:2602. doi: 10.1038/s41467-022-30352-1 (PMC9095838; doi:10.1038/s41467-022-30352-1)
Supplement: Supplementary file 1 — Supplementary Information [file 41467_2022_30352_MOESM1_ESM.pdf]

# Supplementary Information

## Activation of *Xist* by An Evolutionarily Conserved Function of the KDM5C Demethylase

Milan Kumar Samanta, Srimonta Gayen, Clair Harris, Emily Maclary, Yumie Murata-Nakamura, Rebecca M. Malcore, Robert S. Porter, Patricia M. Garay, Christina N. Vallianatos, Paul B. Samollow, Shigeki Iwase, and Sundeep Kalantry

Supplementary Figure 1. Characterization of $Kdm5c^{\Delta/\Delta}$ mutation.

Supplementary Figure 2. Differentiation regimen of ESCs into EpiLCs.

Supplementary Figure 3. Characterization of transgene expression in $X^{\Delta Tsix}Y$ ESCs.

Supplementary Figure 4. Alignment of therian and platypus KDM5C and KDM5D proteins.

Supplementary Table 1. Statistics for Figure 2b: Xist RNA coating in E5.5 epiblast cells.

Supplementary Table 2. Statistics for Figure 2c: Xist RT-qPCR in E3.5 or E5.5 embryos.

Supplementary Table 3. Statistics for Figure 3c: Xist RNA coating in EpiLCs.

Supplementary Table 4. Statistics for Figure 3d: Xist RT-qPCR in EpiLCs.

Supplementary Table 5. Statistics for Figure 4b: Xist RNA coating detection by FISH after Dox induction in *XY* ESCs ectopically expressing *Kdm5c*/*d* transgenes.

Supplementary Table 6. Statistics for RT-qPCR and RNA FISH in *Kdm5c/d* transgenic and mutant $X^{\Delta Tsix}Y$ male EpiLCs.

Supplementary Table 7. Statistics for Figure 5a: KDM5C peaks at the *Xist* locus in day 2 differentiated $X^{\Delta Tsix}Y$ EpiLCs.

Supplementary Table 8. Statistics for Figure 5b: the levels of histone modifications by ChIP-qPCR of $X^{\Delta Tsix}Y$ ESCs ectopically expressing or deleted for *Kdm5c.*

Supplementary Table 9. Statistics for Figure 8: Xist RNA coating detection by FISH after Dox induction in *XY* ESCs ectopically expressing *Kdm5c*/*d* opossum or platypus transgenes.

**a**

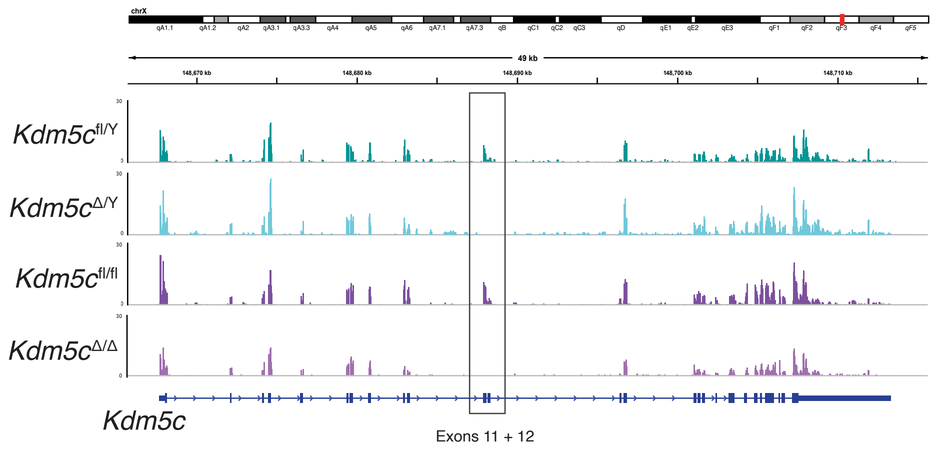


**b**

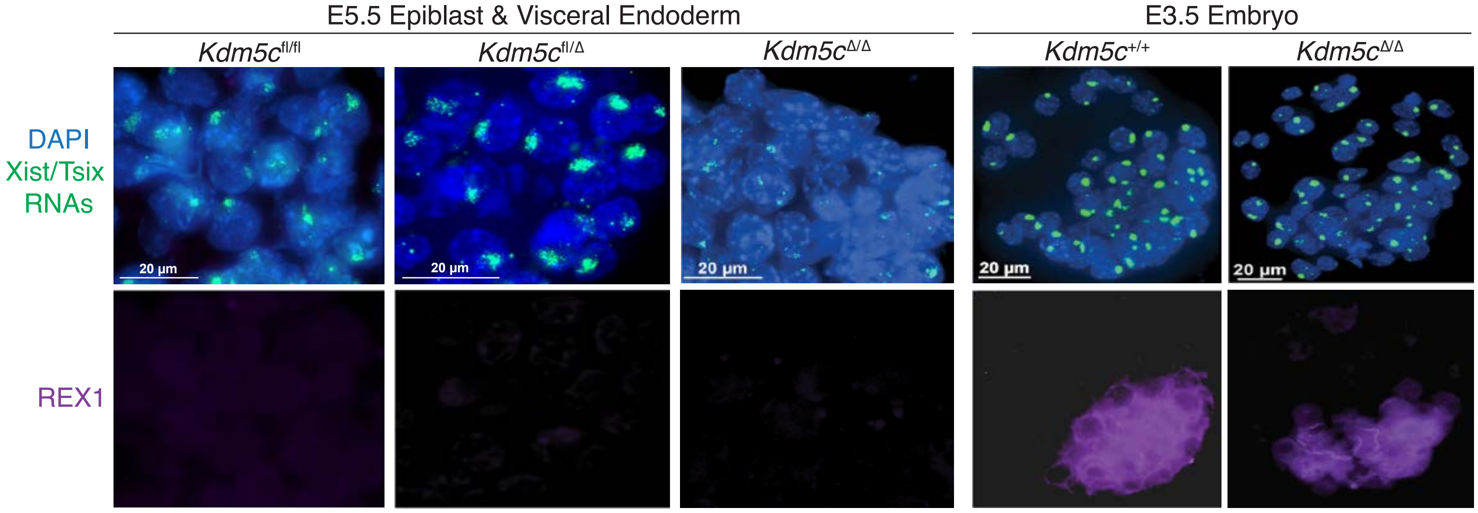


**Supplementary Figure 1. Characterization of *Kdm5c*$^{\Delta/\Delta}$ mutation. (a)** *Kdm5c* expression deduced from RNA-Seq of epiblast-like cells (EpiLCs). *Kdm5c*$^{\Delta}$ allele lacks RNA-Seq reads from *Kdm5c* exons 11 and 12 (boxed), which encode the enzymatic demethylase domain, and produces a barely detectable mutant protein[54]. **(b)** RNA FISH detection of Xist and Tsix RNAs coupled with IF detection of the pluripotency marker REX1 in representative E5.5 embryonic epiblast-enriched cells and E3.5 embryos. REX1 marks pluripotent epiblast progenitor cells and its downregulation is indicative of epiblast differentiation[105]. Nuclei are stained blue with DAPI. RNA FISH was performed on 3 embryos/genotype with similar results.

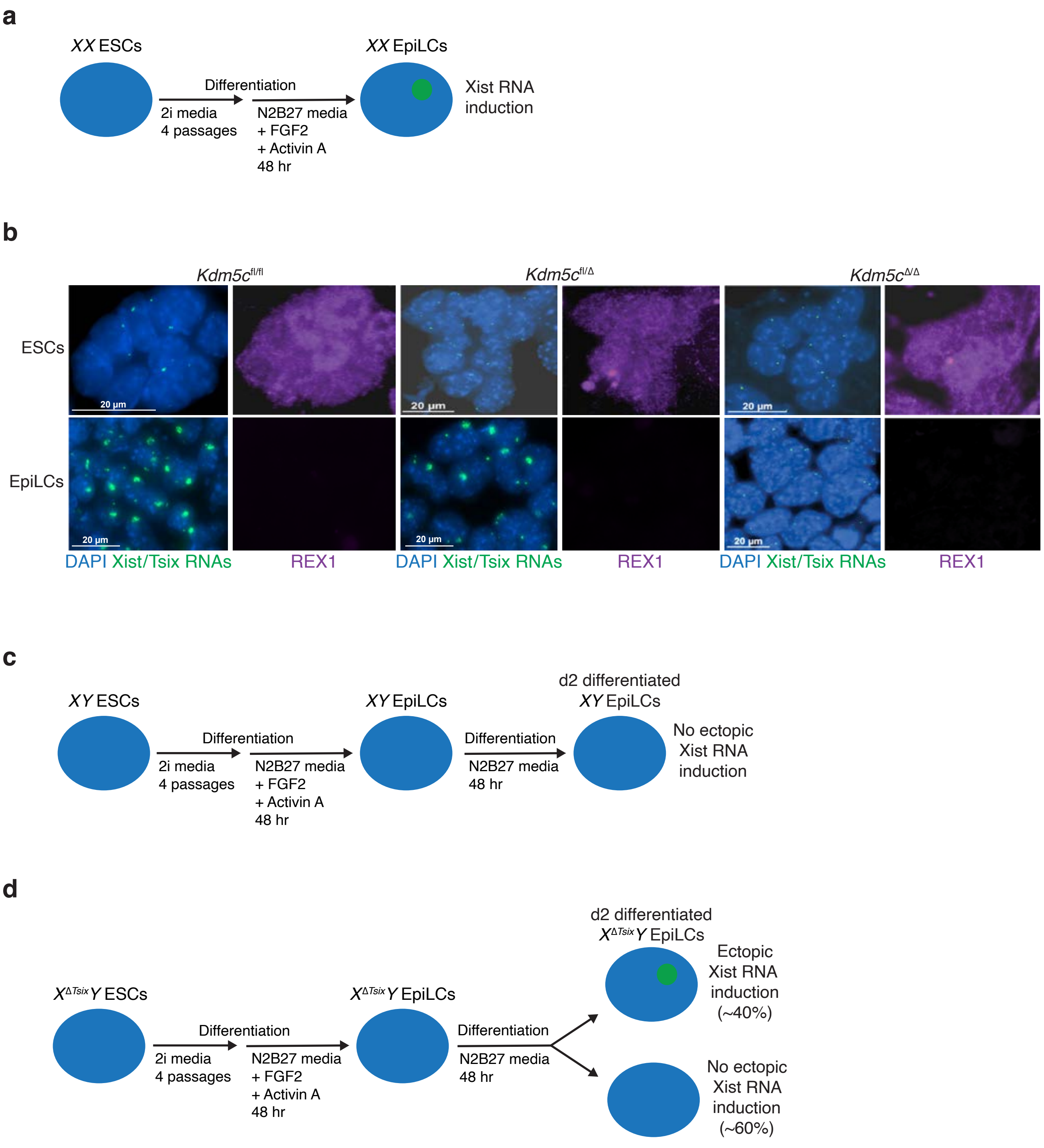


**Supplementary Figure 2. Differentiation regimen of ESCs into EpiLCs. (a)** Differentiation schematic of female ESCs into EpiLCs. **(b)** Xist and Tsix RNAs and REX1 profiling in representative ESCs and EpiLCs. Nuclei are stained blue with DAPI. RNA FISH was performed on three ESC lines/genotype and three ESC lines differentiated into EpiLCs/genotype with similar results. **(c)** Differentiation protocol of *XY* male ESCs into day (d) 2 differentiated EpiLCs. Differentiated wild-type *XY* male EpiLCs do not exhibit Xist RNA coating. **(d)** Differentiation of $X^{\Delta Tsix}Y$ male ESCs into d2 differentiated EpiLCs. A subset of d2 differentiated $X^{\Delta Tsix}Y$ EpiLCs display Xist RNA coating.

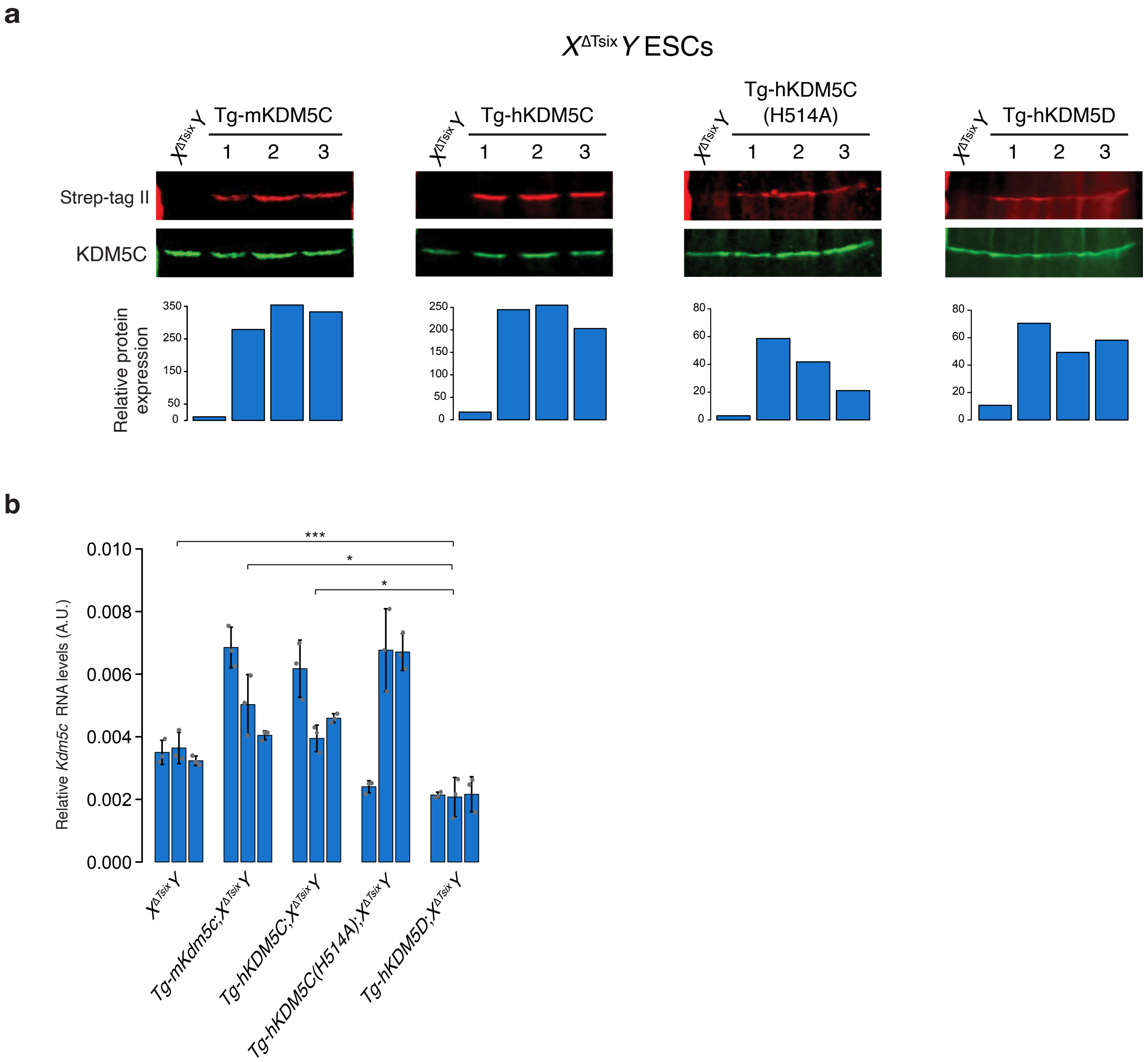


**Supplementary Figure 3. Characterization of transgene expression in *X*$^{\Delta Tsix}$*Y* ESCs. (a)** Analysis of transgenic mouse or human KDM5C/KDM5D protein expression in *X*$^{\Delta Tsix}$*Y* ESCs. Antibody detection of Strep-tag II epitope tagging distinguishes transgenic KDM5C/D from endogenous KDM5C protein by western blotting. Transgenic protein levels were normalized to KDM5C. Each lane is one of three independent ESC lines expressing the indicated transgene. Experiments were performed 3 times with similar results. **(b)** Relative quantification of *Kdm5c* RNA by RT-qPCR in d2 differentiated *X*$^{\Delta Tsix}$*Y* EpiLCs ectopically expressing mouse KDM5C (*Tg-mKdm5c*;*X*$^{\Delta Tsix}$*Y*); human KDM5C (*Tg-hKDM5C*;*X*$^{\Delta Tsix}$*Y*); enzymatically-inactive KDM5C (*Tg-hKDM5C(H514A)*;*X*$^{\Delta Tsix}$*Y*); and, KDM5D (*Tg-hKDM5D*;*X*$^{\Delta Tsix}$*Y*) proteins. Three independent ESC lines were differentiated into d2 differentiated EpiLCs/genotype. Each EpiLC line was analyzed in triplicate. Data are presented as mean ± standard deviation between technical replicates. p-values, Welch's two-sided t-test on ΔCt values of biological replicates. *, $p \leq 0.05$; **, $p < 0.01$; ***, $p < 0.001$. See Supplementary Table 6 for p-values of all pairwise statistical comparisons. Source data and full western blots are provided as a Source Data file.

a

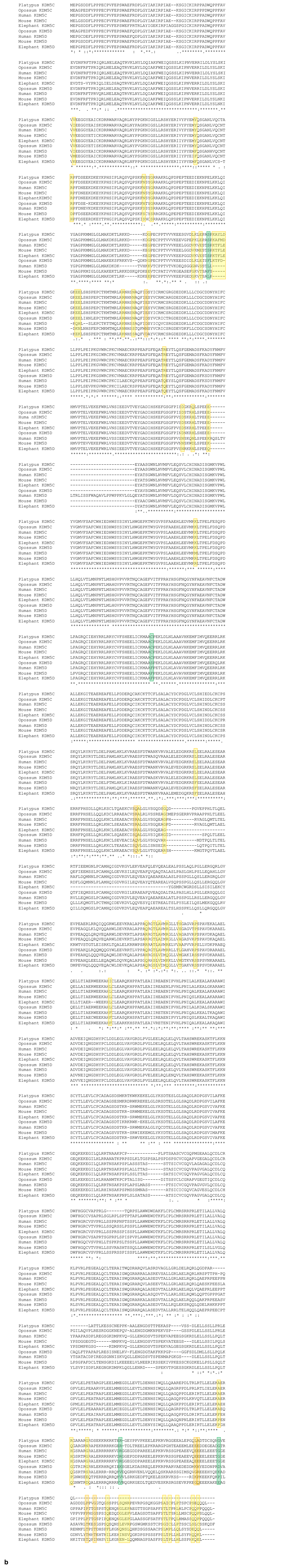


**Supplementary Figure 4. Alignment of therian and platypus KDM5C and KDM5D proteins.** **(a)** Alignment of therian and platypus KDM5C and KDM5D proteins. Amino acids conserved between all eutherian KDM5C and diverged from eutherian KDM5D (45) are highlighted in yellow. Amino acids conserved in therian KDM5C and diverged in therian KDM5D (6) are highlighted in orange. Amino acids conserved in therian and platypus KDM5C proteins and diverged in therian KDM5D (4) are highlighted in green. *, conserved site; :, sites with conservative replacements; ., sites with semi-conservative replacements. **(b)** Domain structure and amino acid positions of mouse KDM5C. Red bars indicate the four amino acids, highlighted in green in **(a)**, conserved in all KDM5C proteins but diverged in KDM5D. Each bar is labeled with the amino acid and its position in KDM5C and KDM5D of each species.

**Supplementary Table 1.** Statistics for Figure 2b: Xist RNA coating in E5.5 epiblast cells.

p-values from all pairwise comparisons determined by Welch's two-sided t-test.

**E5.5 Epiblast- Large Xist RNA coat**

| | *Kdm5c*$^{fl/\Delta}$ | *Kdm5c*$^{\Delta/\Delta}$ | *Kdm5c*$^{fl}$*Y* | *Kdm5c*$^{\Delta}$*Y* |
|---|---|---|---|---|
| *Kdm5c*$^{fl/fl}$ | 0.117 | 0.000 | 0.002 | 0.002 |
| *Kdm5c*$^{fl/\Delta}$ | | 0.002 | 0.003 | 0.003 |
| *Kdm5c*$^{\Delta/\Delta}$ | | | 0.006 | 0.006 |

**E5.5 Epiblast- Small Xist RNA coat**

| | *Kdm5c*$^{fl/\Delta}$ | *Kdm5c*$^{\Delta/\Delta}$ | *Kdm5c*$^{fl}$*Y* | *Kdm5c*$^{\Delta}$*Y* |
|---|---|---|---|---|
| *Kdm5c*$^{fl/fl}$ | 0.313 | 0.728 | 0.020 | 0.020 |
| *Kdm5c*$^{fl/\Delta}$ | | 0.237 | 0.128 | 0.128 |
| *Kdm5c*$^{\Delta/\Delta}$ | | | 0.014 | 0.014 |

**E5.5 Epiblast- Large dispersed Xist RNA coat**

| | *Kdm5c*$^{fl/\Delta}$ | *Kdm5c*$^{\Delta/\Delta}$ | *Kdm5c*$^{fl}$*Y* | *Kdm5c*$^{\Delta}$*Y* |
|---|---|---|---|---|
| *Kdm5c*$^{fl/fl}$ | 0.442 | 0.250 | 0.009 | 0.009 |
| *Kdm5c*$^{fl/\Delta}$ | | 0.177 | 0.070 | 0.070 |
| *Kdm5c*$^{\Delta/\Delta}$ | | | 0.012 | 0.012 |

**E5.5 Epiblast- Small dispersed Xist RNA coat**

| | *Kdm5c*$^{fl/\Delta}$ | *Kdm5c*$^{\Delta/\Delta}$ | *Kdm5c*$^{fl}$*Y* | *Kdm5c*$^{\Delta}$*Y* |
|---|---|---|---|---|
| *Kdm5c*$^{fl/fl}$ | 0.436 | 0.082 | 0.184 | 0.184 |
| *Kdm5c*$^{fl/\Delta}$ | | 0.017 | 0.017 | 0.017 |
| *Kdm5c*$^{\Delta/\Delta}$ | | | 0.004 | 0.004 |

**E5.5 Epiblast- No Xist RNA coating**

| | *Kdm5c*$^{fl/\Delta}$ | *Kdm5c*$^{\Delta/\Delta}$ | *Kdm5c*$^{fl}$*Y* | *Kdm5c*$^{\Delta}$*Y* |
|---|---|---|---|---|
| *Kdm5c*$^{fl/fl}$ | 0.084 | 0.003 | 0.000 | 0.000 |
| *Kdm5c*$^{fl/\Delta}$ | | 0.157 | 0.002 | 0.002 |
| *Kdm5c*$^{\Delta/\Delta}$ | | | 0.000 | 0.000 |

p-values from Chi-Square analysis between genotypes for all categories of Xist RNA coating in E5.5 Epiblast cells.

**E5.5 Epiblast- All categories of Xist RNA coating**

| | *Kdm5c*$^{fl/\Delta}$ | *Kdm5c*$^{\Delta/\Delta}$ |
|---|---|---|
| *Kdm5c*$^{fl/fl}$ | 2.11E-06 | 5.64E-16 |
| *Kdm5c*$^{fl/\Delta}$ | | 1.06E-07 |

**Supplementary Table 2.** Statistics for Figure 2c: Xist RT-qPCR in E3.5 or E5.5 embryos.

p-values from all pairwise comparisons determined by Welch's two-sided t-test.

## E5.5 Epiblast

| | *Kdm5c*$^{fl/\Delta}$ | *Kdm5c*$^{\Delta/\Delta}$ | *Kdm5c*$^{fl}$*Y* | *Kdm5c*$^{\Delta}$*Y* |
|---|---|---|---|---|
| *Kdm5c*$^{fl/fl}$ | 0.894 | 0.003 | 0.208 | 0.091 |
| *Kdm5c*$^{fl/\Delta}$ | | 0.003 | 0.206 | 0.072 |
| *Kdm5c*$^{\Delta/\Delta}$ | | | 0.263 | 0.266 |
| *Kdm5c*$^{fl}$*Y* | | | | 0.299 |

## E5.5 Extraembryonic Ectoderm

| | *Kdm5c*$^{fl/\Delta}$ | *Kdm5c*$^{\Delta/\Delta}$ | *Kdm5c*$^{fl}$*Y* | *Kdm5c*$^{\Delta}$*Y* |
|---|---|---|---|---|
| *Kdm5c*$^{fl/fl}$ | 0.721 | 0.141 | 0.106 | 0.080 |
| *Kdm5c*$^{fl/\Delta}$ | | 0.506 | 0.103 | 0.070 |
| *Kdm5c*$^{\Delta/\Delta}$ | | | 0.111 | 0.089 |
| *Kdm5c*$^{fl}$*Y* | | | | 0.274 |

## E3.5 blastocysts

| | *Kdm5c*$^{fl/\Delta}$ | *Kdm5c*$^{\Delta/\Delta}$ |
|---|---|---|
| *Kdm5c*$^{fl/fl}$ | 0.363 | 0.195 |
| *Kdm5c*$^{fl/\Delta}$ | | 0.946 |

**Supplementary Table 3.** Statistics for Figure 3c: Xist RNA coating in EpiLCs.

p-values from all pairwise comparisons determined by Welch's two-sided t-test.

**EpiLCs- Large Xist RNA coat**

| | $Kdm5c^{fl/\Delta}$ | $Kdm5c^{\Delta/\Delta}$ | $Kdm5c^{fl}Y$ | $Kdm5c^{\Delta}Y$ |
|---|---|---|---|---|
| $Kdm5c^{fl/fl}$ | 0.375 | 0.005 | 0.006 | 0.006 |
| $Kdm5c^{fl/\Delta}$ | | 0.010 | 0.006 | 0.006 |
| $Kdm5c^{\Delta/\Delta}$ | | | 0.071 | 0.089 |
| $Kdm5c^{fl}Y$ | | | | 0.097 |

**EpiLCs- Small Xist RNA coat**

| | $Kdm5c^{fl/\Delta}$ | $Kdm5c^{\Delta/\Delta}$ | $Kdm5c^{fl}Y$ | $Kdm5c^{\Delta}Y$ |
|---|---|---|---|---|
| $Kdm5c^{fl/fl}$ | 0.287 | 0.807 | 0.020 | 0.020 |
| $Kdm5c^{fl/\Delta}$ | | 0.096 | 0.005 | 0.006 |
| $Kdm5c^{\Delta/\Delta}$ | | | 0.000 | 0.002 |
| $Kdm5c^{fl}Y$ | | | | 0.184 |

**EpiLCs - Large dispersed Xist RNA coat**

| | $Kdm5c^{fl/\Delta}$ | $Kdm5c^{\Delta/\Delta}$ | $Kdm5c^{fl}Y$ | $Kdm5c^{\Delta}Y$ |
|---|---|---|---|---|
| $Kdm5c^{fl/fl}$ | 0.045 | 0.074 | 0.022 | 0.040 |
| $Kdm5c^{fl/\Delta}$ | | 0.450 | 0.001 | 0.003 |
| $Kdm5c^{\Delta/\Delta}$ | | | 0.022 | 0.029 |
| $Kdm5c^{fl}Y$ | | | | 0.449 |

**EpiLCs - Small dispersed Xist RNA coat**

| | $Kdm5c^{fl/\Delta}$ | $Kdm5c^{\Delta/\Delta}$ | $Kdm5c^{fl}Y$ | $Kdm5c^{\Delta}Y$ |
|---|---|---|---|---|
| $Kdm5c^{fl/fl}$ | 0.157 | 0.015 | 0.003 | 0.001 |
| $Kdm5c^{fl/\Delta}$ | | 0.006 | 0.009 | 0.006 |
| $Kdm5c^{\Delta/\Delta}$ | | | 0.001 | 0.003 |
| $Kdm5c^{fl}Y$ | | | | 0.358 |

**EpiLCs - No Xist RNA coating**

| | $Kdm5c^{fl/\Delta}$ | $Kdm5c^{\Delta/\Delta}$ | $Kdm5c^{fl}Y$ | $Kdm5c^{\Delta}Y$ |
|---|---|---|---|---|
| $Kdm5c^{fl/fl}$ | 0.903 | 0.061 | 0.001 | 0.002 |
| $Kdm5c^{fl/\Delta}$ | | 0.072 | 0.000 | 0.000 |
| $Kdm5c^{\Delta/\Delta}$ | | | 0.002 | 0.005 |
| $Kdm5c^{fl}Y$ | | | | 0.893 |

p-values from Chi-Square analysis between genotypes for all categories of Xist expression in EpiLCs.

**EpiLCs - All categories of Xist RNA coating**

| | $Kdm5c^{fl/\Delta}$ | $Kdm5c^{\Delta/\Delta}$ |
|---|---|---|
| $Kdm5c^{fl/fl}$ | 0.7595 | 7.60E-09 |
| $Kdm5c^{fl/\Delta}$ | | 7.63E-09 |

**Supplementary Table 4.** Statistics for Figure 3d: Xist RT-qPCR in EpiLCs.

p-values from all pairwise comparisons determined by Welch's two-sided t-test.

**EpiLCs**

| | *Kdm5c*$^{fl/\Delta}$ | *Kdm5c*$^{\Delta/\Delta}$ | *Kdm5c*$^{fl}$*Y* | *Kdm5c*$^{\Delta}$*Y* |
|---|---|---|---|---|
| *Kdm5c*$^{fl/fl}$ | 0.193 | 0.001 | 0.009 | 0.007 |
| *Kdm5c*$^{fl/\Delta}$ | | 0.056 | 0.054 | 0.002 |
| *Kdm5c*$^{\Delta/\Delta}$ | | | 0.453 | 0.027 |
| *Kdm5c*$^{fl}$*Y* | | | | 0.012 |

**Supplementary Table 5.** Statistics for Figure 4b: Xist RNA coating detection by FISH after Dox induction in *XY* ESCs ectopically expressing *Kdm5c/d* transgenes.

p-values from all pairwise comparisons determined by Welch's two-sided t-test.

| **HA positive** | **HA positive** | | | |
|---|---|---|---|---|
| | *Tg-mKdm5c* | *Tg-hKDM5C* | *Tg-hKDM5C(H514A)* | *Tg-hKDM5D* |
| *Tg-mKdm5c* | | 0.16147 | 0.00126 | 0.00254 |
| *Tg-hKDM5C* | | | 0.00001 | 0.00008 |
| *Tg-hKDM5C(H514A)* | | | | 0.00228 |

| **HA negative** | **HA positive** | | | |
|---|---|---|---|---|
| | *Tg-mKdm5c* | *Tg-hKDM5C* | *Tg-hKDM5C(H514A)* | *Tg-hKDM5D* |
| *Tg-mKdm5c* | | 0.268 | 0.247 | 0.484 |
| *Tg-hKDM5C* | | | 0.970 | 0.414 |
| *Tg-hKDM5C(H514A)* | | | | 0.393 |

| **HA negative** | **HA negative** | | | |
|---|---|---|---|---|
| | *Tg-mKdm5c* | *Tg-hKDM5C* | *Tg-hKDM5C(H514A)* | *Tg-hKDM5D* |
| *Tg-mKdm5c* | 0.00126 | | | |
| *Tg-hKDM5C* | | 0.00010 | | |
| *Tg-hKDM5C(H514A)* | | | 0.84698 | |
| *Tg-hKDM5D* | | | | 0.01214 |

**Supplementary Table 6.** Statistics for RT-qPCR and RNA FISH in *Kdm5c/d* transgenic and mutant $X^{\Delta Tsix}Y$ male EpiLCs.

p-values from all pairwise comparisons determined by Welch's two-sided t-test.

## Figure 4d Xist RT-qPCR

| | *Tg-mKDM5C; $X^{\Delta Tsix}Y$* | *Tg-hKDM5C; $X^{\Delta Tsix}Y$* | *Tg-hKDM5C(H514A); $X^{\Delta Tsix}Y$* |
|---|---|---|---|
| $X^{\Delta Tsix}Y$ | 0.0316 | 0.0541 | 0.6273 |
| *Tg-mKDM5C;$X^{\Delta Tsix}Y$* | | 0.8882 | 0.0312 |
| *Tg-hKDM5C;$X^{\Delta Tsix}Y$* | | | 0.0562 |

| | *Tg-hKDM5D; $X^{\Delta Tsix}Y$* | $X^{\Delta Tsix;\Delta Kdm5c}Y$ | *XX* | *XY* |
|---|---|---|---|---|
| $X^{\Delta Tsix}Y$ | 0.9726 | 0.0118 | 0.0000 | 0.0004 |
| *Tg-mKDM5C;$X^{\Delta Tsix}Y$* | 0.0360 | 0.0011 | 0.0092 | 0.0000 |
| *Tg-hKDM5C;$X^{\Delta Tsix}Y$* | 0.0574 | 0.0011 | 0.0199 | 0.0000 |
| *Tg-hKDM5C(H514A);$X^{\Delta Tsix}Y$* | 0.5800 | 0.0093 | 0.0000 | 0.0003 |
| *Tg-hKDM5D;$X^{\Delta Tsix}Y$* | | 0.0135 | 0.0000 | 0.0005 |
| $X^{\Delta Tsix;\Delta Kdm5c}Y$ | | | 0.0022 | 0.0000 |
| *XX* | | | | 0.0003 |

## Figure 4d Xist RNA Coating

| | *Tg-mKDM5C; $X^{\Delta Tsix}Y$* | *Tg-hKDM5C; $X^{\Delta Tsix}Y$* | *Tg-hKDM5C(H514A); $X^{\Delta Tsix}Y$* |
|---|---|---|---|
| $X^{\Delta Tsix}Y$ | 0.0007 | 0.0026 | 0.7453 |
| *Tg-mKDM5C;$X^{\Delta Tsix}Y$* | | 0.0330 | 0.0005 |
| *Tg-hKDM5C;$X^{\Delta Tsix}Y$* | | | 0.0015 |
| *Tg-hKDM5C(H514A);$X^{\Delta Tsix}Y$* | | | |

| | *Tg-hKDM5D; $X^{\Delta Tsix}Y$* | $X^{\Delta Tsix;\Delta Kdm5c}Y$ |
|---|---|---|
| $X^{\Delta Tsix}Y$ | 0.8060 | 0.0033 |
| *Tg-mKDM5C;$X^{\Delta Tsix}Y$* | 0.0004 | 0.0000 |
| *Tg-hKDM5C;$X^{\Delta Tsix}Y$* | 0.0004 | 0.0001 |
| *Tg-hKDM5C(H514A);$X^{\Delta Tsix}Y$* | 0.4994 | 0.0016 |

## Supplementary Figure 3b *Kdm5c* RT-qPCR

| | *Tg-mKDM5C*; $X^{\Delta Tsix}Y$ | *Tg-hKDM5C;* $X^{\Delta Tsix}Y$ | *Tg-hKDM5C(H514A);* $X^{\Delta Tsix}Y$ | *Tg-hKDM5D;* $X^{\Delta Tsix}Y$ |
|---|---|---|---|---|
| $X^{\Delta Tsix}Y$ | 0.11 | 0.12 | 0.46 | 0.0007 |
| *Tg-mKDM5C*;$X^{\Delta Tsix}Y$ | | 0.74 | 0.81 | 0.03 |
| *Tg-hKDM5C;*$X^{\Delta Tsix}Y$ | | | 0.95 | 0.02 |
| *Tg-hKDM5C(H514A);*$X^{\Delta Tsix}Y$ | | | | 0.14 |

**Supplementary Table 7.** Statistics for Figure 5a: KDM5C peaks at the *Xist* locus in day 2 differentiated $X^{\Delta Tsix}Y$ EpiLCs.

p-values from all pairwise comparisons determined by Welch's two-sided t-test.

| Scaling factor: | 1 | 1.269 | 0.782 | 16.179 |
|---|---|---|---|---|
| | $X^{\Delta Tsix}Y$ Replicate 1 | $X^{\Delta Tsix}Y$ Replicate 2 | $X^{\Delta Tsix;\Delta Kdm5c}Y$ Replicate 1 | $X^{\Delta Tsix;\Delta Kdm5c}Y$ Replicate 2 |
| KDM5C reads | 25 | 41 | 10 | 35 |
| KDM5C normalized reads | 25 | 32.297 | 12.793 | 2.163 |
| | $X^{\Delta Tsix}Y$ | | $X^{\Delta Tsix;\Delta Kdm5c}Y$ | |
| Average KDM5C normalized reads | 28.6 | | 7.5 | |

**Supplementary Table 8.** Statistics for Figure 5b: the levels of histone modifications by ChIP-qPCR of $X^{\Delta Tsix}Y$ ESCs ectopically expressing or deleted for *Kdm5c.*

p-values from all pairwise comparisons determined by Welch's two-sided t-test.

## Region 1: KDM5C-enriched region downstream of *Xist* TSS

### H3K4me1

| | *Tg-hKDM5C;*$X^{\Delta Tsix}Y$ | *Tg-hKDM5C(H514A);*$X^{\Delta Tsix}Y$ | $X^{\Delta Tsix;\Delta Kdm5c}Y$ |
|---|---|---|---|
| $X^{\Delta Tsix}Y$ | 0.0072 | 0.7715 | 0.0415 |
| *Tg-hKDM5C;*$X^{\Delta Tsix}Y$ | | 0.0085 | 0.0016 |
| *Tg-hKDM5C(H514A);*$X^{\Delta Tsix}Y$ | | | 0.0282 |

### H3K4me2

| | *Tg-hKDM5C;*$X^{\Delta Tsix}Y$ | *Tg-hKDM5C(H514A);*$X^{\Delta Tsix}Y$ | $X^{\Delta Tsix;\Delta Kdm5c}Y$ |
|---|---|---|---|
| $X^{\Delta Tsix}Y$ | 0.0803 | 0.6720 | 0.1562 |
| *Tg-hKDM5C;*$X^{\Delta Tsix}Y$ | | 0.0221 | 0.0360 |
| *Tg-hKDM5C(H514A);*$X^{\Delta Tsix}Y$ | | | 0.1205 |

### H3K4me3

| | *Tg-hKDM5C;*$X^{\Delta Tsix}Y$ | *Tg-hKDM5C(H514A);*$X^{\Delta Tsix}Y$ | $X^{\Delta Tsix;\Delta Kdm5c}Y$ |
|---|---|---|---|
| $X^{\Delta Tsix}Y$ | 0.0384 | 0.2428 | 0.4435 |
| *Tg-hKDM5C;*$X^{\Delta Tsix}Y$ | | 0.0052 | 0.1571 |
| *Tg-hKDM5C(H514A);*$X^{\Delta Tsix}Y$ | | | 0.2904 |

### H3K27ac

| | *Tg-hKDM5C;*$X^{\Delta Tsix}Y$ | *Tg-hKDM5C(H514A);*$X^{\Delta Tsix}Y$ | $X^{\Delta Tsix;\Delta Kdm5c}Y$ |
|---|---|---|---|
| $X^{\Delta Tsix}Y$ | 0.0258 | 0.5022 | 0.9212 |
| *Tg-hKDM5C;*$X^{\Delta Tsix}Y$ | | 0.0376 | 0.0372 |
| *Tg-hKDM5C(H514A);*$X^{\Delta Tsix}Y$ | | | 0.3477 |

### IgG

| | *Tg-hKDM5C;*$X^{\Delta Tsix}Y$ | *Tg-hKDM5C(H514A);*$X^{\Delta Tsix}Y$ | $X^{\Delta Tsix;\Delta Kdm5c}Y$ |
|---|---|---|---|
| $X^{\Delta Tsix}Y$ | 0.9572 | 0.6574 | 0.3226 |
| *Tg-hKDM5C;*$X^{\Delta Tsix}Y$ | | 0.6914 | 0.3386 |
| *Tg-hKDM5C(H514A);*$X^{\Delta Tsix}Y$ | | | 0.3174 |

## **Region 2: *Xist* promoter**

### H3K4me1

| | *Tg-hKDM5C;*$X^{\Delta Tsix}Y$ | *Tg-hKDM5C(H514A);*$X^{\Delta Tsix}Y$ | $X^{\Delta Tsix;\Delta Kdm5c}Y$ |
|---|---|---|---|
| $X^{\Delta Tsix}Y$ | 0.7419 | 0.8437 | 0.4762 |
| *Tg-hKDM5C;*$X^{\Delta Tsix}Y$ | | 0.4556 | 0.4942 |
| *Tg-hKDM5C(H514A);*$X^{\Delta Tsix}Y$ | | | 0.1850 |

### H3K4me2

| | *Tg-hKDM5C;*$X^{\Delta Tsix}Y$ | *Tg-hKDM5C(H514A);*$X^{\Delta Tsix}Y$ | $X^{\Delta Tsix;\Delta Kdm5c}Y$ |
|---|---|---|---|
| $X^{\Delta Tsix}Y$ | 0.3324 | 0.4415 | 0.1267 |
| *Tg-hKDM5C;*$X^{\Delta Tsix}Y$ | | 0.4140 | 0.2163 |
| *Tg-hKDM5C(H514A);*$X^{\Delta Tsix}Y$ | | | 0.0719 |

### H3K4me3

| | *Tg-hKDM5C;*$X^{\Delta Tsix}Y$ | *Tg-hKDM5C(H514A);*$X^{\Delta Tsix}Y$ | $X^{\Delta Tsix;\Delta Kdm5c}Y$ |
|---|---|---|---|
| $X^{\Delta Tsix}Y$ | 0.2721 | 0.9119 | 0.0543 |
| *Tg-hKDM5C;*$X^{\Delta Tsix}Y$ | | 0.3190 | 0.1339 |
| *Tg-hKDM5C(H514A);*$X^{\Delta Tsix}Y$ | | | 0.2651 |

### H3K27ac

| | *Tg-hKDM5C;*$X^{\Delta Tsix}Y$ | *Tg-hKDM5C(H514A);*$X^{\Delta Tsix}Y$ | $X^{\Delta Tsix;\Delta Kdm5c}Y$ |
|---|---|---|---|
| $X^{\Delta Tsix}Y$ | 0.0050 | 0.2301 | 0.3364 |
| *Tg-hKDM5C;*$X^{\Delta Tsix}Y$ | | 0.0088 | 0.0071 |
| *Tg-hKDM5C(H514A);*$X^{\Delta Tsix}Y$ | | | 0.1053 |

### IgG

| | *Tg-hKDM5C;*$X^{\Delta Tsix}Y$ | *Tg-hKDM5C(H514A);*$X^{\Delta Tsix}Y$ | $X^{\Delta Tsix;\Delta Kdm5c}Y$ |
|---|---|---|---|
| $X^{\Delta Tsix}Y$ | 0.2889 | 0.0059 | 0.2068 |
| *Tg-hKDM5C;*$X^{\Delta Tsix}Y$ | | 0.0131 | 0.2512 |
| *Tg-hKDM5C(H514A);*$X^{\Delta Tsix}Y$ | | | 0.8027 |

## Region 3: Upstream of *Xist*

### H3K4me1

| | *Tg-hKDM5C;$X^{\Delta Tsix}$Y* | *Tg-hKDM5C(H514A);$X^{\Delta Tsix}$Y* | *$X^{\Delta Tsix;\Delta Kdm5c}$Y* |
|---|---|---|---|
| *$X^{\Delta Tsix}$Y* | 0.1693 | 0.1608 | 0.5787 |
| *Tg-hKDM5C;$X^{\Delta Tsix}$Y* | | 0.7944 | 0.0573 |
| *Tg-hKDM5C(H514A);$X^{\Delta Tsix}$Y* | | | 0.0681 |

### H3K4me2

| | *Tg-hKDM5C;$X^{\Delta Tsix}$Y* | *Tg-hKDM5C(H514A);$X^{\Delta Tsix}$Y* | *$X^{\Delta Tsix;\Delta Kdm5c}$Y* |
|---|---|---|---|
| *$X^{\Delta Tsix}$Y* | 0.0056 | 0.0628 | 0.0274 |
| *Tg-hKDM5C;$X^{\Delta Tsix}$Y* | | 0.5136 | 0.9114 |
| *Tg-hKDM5C(H514A);$X^{\Delta Tsix}$Y* | | | 0.5797 |

### H3K4me3

| | *Tg-hKDM5C;$X^{\Delta Tsix}$Y* | *Tg-hKDM5C(H514A);$X^{\Delta Tsix}$Y* | *$X^{\Delta Tsix;\Delta Kdm5c}$Y* |
|---|---|---|---|
| *$X^{\Delta Tsix}$Y* | 0.9288 | 0.2482 | 0.0531 |
| *Tg-hKDM5C;$X^{\Delta Tsix}$Y* | | 0.2688 | 0.0366 |
| *Tg-hKDM5C(H514A);$X^{\Delta Tsix}$Y* | | | 0.2125 |

### H3K27ac

| | *Tg-hKDM5C;$X^{\Delta Tsix}$Y* | *Tg-hKDM5C(H514A);$X^{\Delta Tsix}$Y* | *$X^{\Delta Tsix;\Delta Kdm5c}$Y* |
|---|---|---|---|
| *$X^{\Delta Tsix}$Y* | 0.2134 | 0.3330 | 0.3951 |
| *Tg-hKDM5C;$X^{\Delta Tsix}$Y* | | 0.2023 | 0.1156 |
| *Tg-hKDM5C(H514A);$X^{\Delta Tsix}$Y* | | | 0.4105 |

### IgG

| | *Tg-hKDM5C;$X^{\Delta Tsix}$Y* | *Tg-hKDM5C(H514A);$X^{\Delta Tsix}$Y* | *$X^{\Delta Tsix;\Delta Kdm5c}$Y* |
|---|---|---|---|
| *$X^{\Delta Tsix}$Y* | 0.6546 | 0.0378 | 0.6610 |
| *Tg-hKDM5C;$X^{\Delta Tsix}$Y* | | 0.1626 | 0.9180 |
| *Tg-hKDM5C(H514A);$X^{\Delta Tsix}$Y* | | | 0.3076 |

**Supplementary Table 9.** Statistics for Figure 8: Xist RNA coating detection by FISH after Dox induction in *XY* ESCs ectopically expressing *Kdm5c/d* opossum or platypus transgenes.

p-values from all pairwise comparisons determined by Welch's two-sided t-test.

## Figure 8a. Opossum transgene expression

| | **HA positive** | |
|---|---|---|
| **HA positive** | *Tg-oKDM5C(H514A)* | *Tg-oKDM5D* |
| *Tg-oKDM5C* | 0.003 | 0.002 |
| *Tg-oKDM5C(H514A)* | | 0.068 |
| | | |

| | **HA positive** | |
|---|---|---|
| **HA negative** | *Tg-oKDM5C(H514A)* | *Tg-oKDM5D* |
| *Tg-oKDM5C* | 0.604 | 0.102 |
| *Tg-oKDM5C(H514A)* | | 0.541 |
| | | |

| | **HA negative** | | |
|---|---|---|---|
| **HA negative** | *Tg-oKDM5C* | *Tg-oKDM5C(H514A)* | *Tg-oKDM5D* |
| *Tg-oKDM5C* | 0.003 | | |
| *Tg-oKDM5C(H514A)* | | 0.310 | |
| *Tg-oKDM5D* | | | 0.080 |

## Figure 8b. Platypus transgene expression

| | **HA negative** |
|---|---|
| **HA positive** | *Tg-pKDM5C* |
| *Tg-pKDM5C* | 0.00001 |
| | |
| | |
