# Supplementary material for: Activation of Xist by an evolutionarily conserved function of KDM5C demethylase
Source: Nat Commun. 2022 May 11;13:2602. doi: 10.1038/s41467-022-30352-1 (PMC9095838; doi:10.1038/s41467-022-30352-1)
Supplement: Supplementary file 2 — Description of additional Supplementary File [file 41467_2022_30352_MOESM2_ESM.pdf]

Descriptions of additional Supplementary Data Files

Supplementary Data 1. RNA-seq samples used for Kdm5c expression analysis across theria.

Supplementary Data 2. KDM5C/Kdm5c TPM values in all species.

Supplementary Data 3-12. TPM values of all genes in all 10 species analysed in Figure 6.

Supplementary Data 13. Description of all Cell lines used in this study.
