# Supplementary material for: Activation of Xist by an evolutionarily conserved function of KDM5C demethylase
Source: Nat Commun. 2022 May 11;13:2602. doi: 10.1038/s41467-022-30352-1 (PMC9095838; doi:10.1038/s41467-022-30352-1)
Supplement: Supplementary file 5 — Reporting Summary [file 41467_2022_30352_MOESM5_ESM.pdf]

nature research

Corresponding author(s): Sundeep Kalantry

Last updated by author(s): April 4, 2022

Reporting Summary

Nature Research wishes to improve the reproducibility of the work that we publish. This form provides structure for consistency and transparency in reporting. For further information on Nature Research policies, see our Editorial Policies and the Editorial Policy Checklist.

Statistics

For all statistical analyses, confirm that the following items are present in the figure legend, table legend, main text, or Methods section.

n/a	Confirmed	
☐	☒	The exact sample size (*n*) for each experimental group/condition, given as a discrete number and unit of measurement
☐	☒	A statement on whether measurements were taken from distinct samples or whether the same sample was measured repeatedly
☐	☒	The statistical test(s) used AND whether they are one- or two-sided<br>*Only common tests should be described solely by name; describe more complex techniques in the Methods section.*
☐	☒	A description of all covariates tested
☐	☒	A description of any assumptions or corrections, such as tests of normality and adjustment for multiple comparisons
☐	☒	A full description of the statistical parameters including central tendency (e.g. means) or other basic estimates (e.g. regression coefficient) AND variation (e.g. standard deviation) or associated estimates of uncertainty (e.g. confidence intervals)
☐	☒	For null hypothesis testing, the test statistic (e.g. *F*, *t*, *r*) with confidence intervals, effect sizes, degrees of freedom and *P* value noted<br>*Give P values as exact values whenever suitable.*
☒	☐	For Bayesian analysis, information on the choice of priors and Markov chain Monte Carlo settings
☒	☐	For hierarchical and complex designs, identification of the appropriate level for tests and full reporting of outcomes
☒	☐	Estimates of effect sizes (e.g. Cohen's *d*, Pearson's *r*), indicating how they were calculated

Our web collection on statistics for biologists contains articles on many of the points above.

Software and code

Policy information about availability of computer code

Data collection	Fluorescent images were taken on the NIS-Elements software version 5.20.02.<br>Fluorescent western blots were imaged using Image Studio Lite from Li-COR, version 5.2.5.<br>qPCR reactions were run with Eppendorf Realplex 2.2 software version 2.2.0.84.
Data analysis	FastQC v0.11.8 for quality control of RNA Seq reads. Picard v2/8/1 was used for the VCFtools command for generation of the allele specific reference genomes. STAR v2.5.2a was used for mapping RNA seq reads in Kdm5c analysis accross therians. Tophat v2.1.0 was used for mapping RNA seq reads in EpiLCs. Samtools v0.1.19 was used for sequencing analysis. Python v2.7 was used for the HTseq command for read counting. Bowtie v1.2.2 was used for ChIP seq mapping. MACS2 v2.1.0.20140616 was used for ChIP seq peak calling. ClustalW v2.1 was used for multiple sequence alignments of Kdm5C and KDM5D protein sequences. MSAViewer version 1.12.0 tree tool was used to create a rooted phylogenetic tree. Chi-square analysis of RNA FISH was performed using R version 4.1.2.

For manuscripts utilizing custom algorithms or software that are central to the research but not yet described in published literature, software must be made available to editors and reviewers. We strongly encourage code deposition in a community repository (e.g. GitHub). See the Nature Research guidelines for submitting code & software for further information.

Data

Policy information about availability of data

All manuscripts must include a data availability statement. This statement should provide the following information, where applicable:

- Accession codes, unique identifiers, or web links for publicly available datasets
- A list of figures that have associated raw data
- A description of any restrictions on data availability

Sequencing data generated for this study have been submitted to the NCBI Gene Expression Omnibus (GEO; http://www.ncbi.nlm.nih.gov/geo/) under accession

number GSE96797. Data in Figure 6 was analyzed from published data available under accession numbers GSE30352 and GSE50747 and project accession number PRJNA591380, raw data from these analyses are available in Supplementary Tables 1-12.

Field-specific reporting

Please select the one below that is the best fit for your research. If you are not sure, read the appropriate sections before making your selection.

[x] Life sciences [ ] Behavioural & social sciences [ ] Ecological, evolutionary & environmental sciences

For a reference copy of the document with all sections, see nature.com/documents/nr-reporting-summary-flat.pdf

Life sciences study design

All studies must disclose on these points even when the disclosure is negative.

Sample size	We followed the convention of field to determine the samples sizes based on our prior publications (Gayen et al., 2016 PNAS; Gayen et al., 2015 Cell Reports). For cell lines, we typically used three independent cell lines for any given genotype as biological replicates.
Data exclusions	We reported all measurements we made without excluding data.
Replication	We carried out experiments with biological triplicates to ensure the reproducibility. For RT-qPCRs and qPCRs, we performed three technical replicates for each biological replicate. For FISH on cell lines, each experiment was performed 3 times on each biological replicate. For FISH on embryos, each embryo can only be stained once so only one experiment was performed for each embryo presented. ChIP experiment was performed 2 times.
Randomization	We analyzed animals, embryos, and cell lines with pre-determined genotypes. Samples were allocated into groups based on their genotype. Multiple cell lines were generated, or embryos collected, from different litters to account for litter to litter variation.
Blinding	For embryos: We were blinded to embryo samples we collected in the study, and we only retroactively genotyped the embryos. Investigators were blinded to group allocation during data collection and analysis. For cell lines: We were not blinded to the samples during culture, but investigators were blinded to group allocation during data collection and analysis.

Reporting for specific materials, systems and methods

We require information from authors about some types of materials, experimental systems and methods used in many studies. Here, indicate whether each material, system or method listed is relevant to your study. If you are not sure if a list item applies to your research, read the appropriate section before selecting a response.

Materials & experimental systems

n/a	Involved in the study
[ ]	[x] Antibodies
[ ]	[x] Eukaryotic cell lines
[x]	[ ] Palaeontology and archaeology
[ ]	[x] Animals and other organisms
[x]	[ ] Human research participants
[x]	[ ] Clinical data
[x]	[ ] Dual use research of concern

Methods

n/a	Involved in the study
[ ]	[x] ChIP-seq
[x]	[ ] Flow cytometry
[x]	[ ] MRI-based neuroimaging

Antibodies

Antibodies used	In-house rabbit anti-KDM5C antibody<br>Anti-KDM5C antibody (Abcam, #194288)<br>Anti-Strep-Tag II antibody (GenScript, #A01732-100)<br>Anti-REX1 antibody (Thermo Scientific, #PA5-27567, Lot# PE1851908)<br>Anti-HA antibody (Cell Signaling, #C29F4, Lot# 9)<br>Anti-H2Av antibody (Active Motif, 39715)<br>Anti-H3K4me1 antibody (Abcam, #ab8895)<br>Anti-H3K4me2 antibody (Abcam, #ab7766, lot# GR102810-1) or ABfinity Rabbit Oligoclonal antibody (Fisher Scientific, cat# 710796, lot# QL230606).<br>Anti-H3K4me3 antibody (Abcam, #ab8580)<br>H3K27ac antibody (Active Motif, #39135)<br>rabbit IgG antibody (Jackson Immunoresearch, #011-000-003)
Validation	Anti-KDM5C antibody (in-house) was raised in rabbit and affinity purified to the antigen (described in Iwase et al., 2016). This antibody was validated by the absence of ChIP-Seq signals in KDM5C-null cells in this study.

Anti-KDM5C antibody (Abcam, #194288) has been validated for use in immunoprecipitation, ChIP, western blotting, and immunohistochemistry as stated on the Abcam product page. Although Abcam states it may detect KDM5D, we validated it's specificity for KDM5C by western blot in mouse ESCs WT or mutant for Kdm5c and/or Kdm5d.
Anti-Strep-Tag II (GenScript, #A01732-100) has been validated for use in immunohistochemistry, western blot, immunoprecipitation, ELISA, and flow cytometry, as stated on the GenScript product page.
Anti-REX1 (Thermo Scientific, #PA5-27567) has been validated for use in immunocytochemistry and western blotting, as stated on the Thermo Scientific product page. This antibody was also validated in this study by use of cells not expressing REX1 as a control.
Anti-HA (Cell Signaling, #C29F4) has been validated for use in western blotting, immunoprecipitation, immunohistochemistry, immunofluorescence, and flow cytometry, as stated on the Cell Signaling Technology product page. This antibody was also validated in this study by use of cells not expressing HA as a control.
Anti-H2Av (Active Motif, #39715) has been validated for chromatin immunoprecipitation and western blotting, as stated on the Active Motif product page.
Anti-H3K4me1 (Abcam, #ab8895) has been validated for use in immunohistochemistry, chromatin immunoprecipitation, and western blotting, as stated on the Abcam product page.
Anti-H3K4me2 (Abcam, #ab7766) has been validated for chromatin immunoprecipitation and western blotting, as stated on the Abcam product page.
Anti-H3K4me2 (Fisher Scientific, #710796) has been validated for chromatin immunoprecipitation, immunofluorescence, peptide array, western blot, and immunocytochemistry, as stated on the Fisher Scientific product page.
Anti-H3K4me3 (Abcam, #ab8580) has been validated for use in chromatin immunoprecipitation, western blotting, immunohistochemistry, and immunofluorescence, as stated on the Abcam product page.
Anti-H3K27ac (Active Motif, #39135) has been validated for use in chromatin immunoprecipitation, western blotting, and CUT&Tag, as stated on the Active Motif product page.
The IgG antibody was validated as a negative control in this study by chromatin immunoprecipitation, and no specific peaks were detected by this antibody.

Eukaryotic cell lines

Policy information about cell lines

Cell line source(s)	Cell lines were derived from mouse embryos for the purposes of this study. HEK2293T cells were purchased from ATCC (#CRL-3216).
Authentication	Cells derived in lab were genotyped and we determined X-chromosome number for each cell line by DNA FISH or by RNA FISH against an X-linked gene expressing from both X-chromosomes. The FISH data are displayed in Figures 3 and 4. HEK cells were not further validated after arrival from ATCC.
Mycoplasma contamination	Cell lines were not tested for Mycoplama contamination.
Commonly misidentified lines (See ICLAC register)	No commonly misidentified lines were used in this study.

Animals and other organisms

Policy information about studies involving animals; ARRIVE guidelines recommended for reporting animal research

Laboratory animals	Animals used in this study were mice of either Mus musculus 129/Sv strain or Mus molossinus JF1 strain. Mice used were either female or male as indicated in the manuscript. Mice were at least 5 weeks of age before use.
Wild animals	The study did not involve wild animals.
Field-collected samples	The study did not involve samples collected from the field.
Ethics oversight	This study was performed in strict accordance with the recommendations in the Guide for the Care and Use of Laboratory Animals of the National Institutes of Health. All animals were handled according to the protocols approved by the University Committee on Use and Care of Animals (UCUCA) at the University of Michigan (protocol #PRO00004007 and PRO00006455).

Note that full information on the approval of the study protocol must also be provided in the manuscript.

ChIP-seq

Data deposition

- [x] Confirm that both raw and final processed data have been deposited in a public database such as GEO.
- [x] Confirm that you have deposited or provided access to graph files (e.g. BED files) for the called peaks.

Data access links *May remain private before publication.*	All ChIP-Seq data are available under GEO accession GSE96740
Files in database submission	KDM5C (named SMCX in the samples below) on the X-chromosome was profiled by chromatin immunoprecipitation (ChIP) in Tsix-deleted, Kdm5c-WT (wild-type) or KO (knock-out or null) embryonic stem cells (ESCs) differentiated for 2 days beyond the epiblast-like cell stage. Two biological replicates of input and ChIP'd sample were sequenced for all genotypes except Kdm5c KO, which only had one replicate for the KDM5C IP. The files are listed below and described in more detail in our GEO

submission:

Smcx_Input_delTsix_wtSmcx_rep1.fastq.gz
Smcx_Input_delTsix_wtSmcx_rep2.fastq.gz
Smcx_Input_delTsix_koSmcx_rep1.fastq.gz
Smcx_IP_delTsix_wtSmcx_rep1.fastq.gz
Smcx_IP_delTsix_wtSmcx_rep2.fastq.gz
Smcx_IP_delTsix_koSmcx_rep1.fastq.gz

Smcx_merged_Input.bw
Smcx_IP_wtSmcx_Merged.bw
Smcx_IP_koSmcx.bw
Smcx_delTsix_wtSmcx_peaks.bed.gz
Smcx_delTsix_koSmcx_peaks.bed.gz

Genome browser session (e.g. UCSC)

No longer applicable.

Methodology

Replicates

Two biological replicates were used. Cells were cultured independently and the entire ChIP procedure and library prep were done independently.

Sequencing depth

Each sample had at least 20,000,000 reads and at least 15,000,000 uniquely mapping reads. Reads were single-ended and 50 bp in length.

Antibodies

H3K4me2 antibody (Abcam #7766, lot# GR102810-1)
KDM5C (named SMCX above) antibody (in-house antibody)
Drosophila anti-H2Av antibody (in-house antibody)

Peak calling parameters

Reads were mapped using bowtie version 2 allowing up to 2 mismatches. We aligned to mm9. Peaks were called using MACS2 software (version 2.1.0.20140616) using input bam files for normalization, with filters for a q-value < 0.1 and a fold enrichment greater than 1. Call line: macs2 callpeak -f BAM -g 1.87e9 -q 0.1 -m 1 150 -s 250 -t "IP File 1" "IP File 2" -c "Input File 1" "Input File 2".

Data quality

Peak calling was performed using input controls to prevent false positives. An FDR of 10% was used to exclude low-confidence peaks. 40% of peaks had >4-fold enrichment. Genome-wide visualization of peaks using Integrated Genome Viewer ensured artifacts were excluded from peak quantification. Peaks of interest were examined visually using Integrated Genome Viewer to validate the enrichment of IP reads over input reads.

Software

For H3K4me2 ChIP-Seq, coverage was calculated using BedTools to calculate read density relative to the Drosophila spike-in. For KDM5C ChIP-Seq analysis, peaks were called using MACS2 software (version 2.1.0.20140616) using input bam files for normalization, with filters for a q-value < 0.1 and a fold enrichment greater than 1.
